# Teach-Back-Based Interventions for Parents and Caregivers in Pediatric Healthcare: A Systematic Review of Randomized Controlled Trials

**DOI:** 10.3390/healthcare14213615

**Published:** 2026-10-26

**Authors:** Şule Şenol, Habibe Ozcelik, Hasan Hüseyin Avcı

**Affiliations:** 1 Department of Child Health and Disease Nursing, Faculty of Nursing, Akdeniz University, Antalya 07058, Türkiye; sulesenol@akdeniz.edu.tr; 2 Department of Public Health Nursing, Faculty of Nursing, Akdeniz University, Antalya 07058, Türkiye; 3 Department of Family Medicine, Faculty of Medicine, Akdeniz University, Antalya 07070, Türkiye; hasanavci@akdeniz.edu.tr

**Keywords:** teach-back, pediatric healthcare, parents, caregivers, health literacy, medication safety, caregiver education, patient education

## Abstract

**Highlights:**

**What are the main findings?**

• Teach-Back-based, multicomponent interventions showed the clearest and most consistent benefits for pediatric medication dosing accuracy and safety (moderate-certainty evidence), while selected improvements in caregivers’ medication or safe-dose knowledge were supported by low-certainty evidence.
• Findings for asthma, oral health, diabetes self-care and glycemic control, and caregiver self-efficacy were more variable and generally supported by low- or very-low-certainty evidence.

**What are the implications of the main findings?**

• Teach-Back may be particularly useful as part of health-literacy-sensitive, multicomponent caregiver education, especially when medication administration and discharge safety are priorities.
• Future pediatric trials should clearly define the core components of Teach-Back, assess intervention fidelity, and use more comparable outcome measures to determine its independent effect and generalizability.

**Abstract:**

**Background/Objectives:** Teach-Back is a closed-loop communication strategy used to verify understanding of health information. This review examined Teach-Back-based interventions for parents and primary caregivers in pediatric healthcare. **Methods:** Following PRISMA 2020 and PRISMA-S, this PROSPERO-registered review (CRD420261504251) searched PubMed/MEDLINE, CENTRAL, CINAHL Ultimate, Web of Science Core Collection, Scopus, ClinicalTrials.gov, and WHO ICTRP without language or publication-year restrictions. Eligible studies were randomized controlled trials involving parents or primary caregivers of children aged 0–18 years. Risk of bias was assessed with RoB 2 and certainty of evidence with GRADE. Owing to clinical and methodological heterogeneity, findings were synthesized narratively. **Results:** Nine randomized controlled trials (1299 participants; individual study sample sizes ranging from 26 to 398) were included. The most consistent benefits were observed for medication dosing accuracy/safety (moderate-certainty evidence) and selected caregiver medication or safe-dose knowledge outcomes (low-certainty evidence). Certainty was low for asthma control and HbA1c and very low for oral health/care performance, asthma-related healthcare utilization, diabetes self-care, and caregiver self-efficacy. No principal-outcome assessment was judged to be at low risk of bias. **Conclusions:** Interventions incorporating Teach-Back may improve selected caregiver outcomes, particularly medication safety and knowledge. However, heterogeneity, multicomponent interventions, and methodological limitations restrict conclusions about the independent effect and generalizability of Teach-Back across pediatric settings.

## 1. Introduction

Parents and primary caregivers are essential to maintaining the continuity and safety of pediatric care, particularly when health-related responsibilities must be carried out at home rather than in a clinical setting. Parental health literacy has been associated with caregiving practices and child health outcomes, including in early childhood and among children with chronic conditions [1,2]. Among children with chronic conditions, lower parental health literacy has been associated with poorer health outcomes, while disease-specific parental knowledge may influence parent health behavior and child outcomes [2]. Current literature on pediatric health literacy emphasizes that, beyond individual health literacy skills, healthcare professionals’ communication style and the healthcare system’s capacity to facilitate access to information are crucial to providing understandable, actionable health information [3].

Effective communication is particularly critical during discharge and other transitions of care. During these periods, caregivers must quickly understand and correctly implement medication regimens, symptom-monitoring recommendations, follow-up plans, and other components of home care. A recent systematic review of medication safety education for parents of pediatric patients reported that structured educational interventions could improve caregivers’ medication knowledge and reduce home medication administration errors. However, studies varied widely in educational methods and intervention components [4]. A randomized study involving caregivers of hospitalized children also demonstrated that discharge communication tailored to health literacy levels could reduce medication dosing errors at home [5]. Therefore, the mere transmission of health information is not a sufficient indicator that the caregiver has understood it; communication methods are needed that enable active verification of understanding.

Teach-Back is a structured, closed-loop communication method used to verify whether health information has been understood correctly [6,7]. In this method, the parent or caregiver restates the information in their own words; when teaching a practical skill, the “Show-Back” approach may be used to verify the skill through demonstration [6]. If gaps or misconceptions are identified, the healthcare professional re-explains the information and re-assesses understanding [6,7]. Thus, Teach-Back aims to evaluate the clarity of the healthcare professional’s communication rather than testing the caregiver’s knowledge or memory [6]. Although definitions of Teach-Back vary in the literature, a systematic review and thematic synthesis have identified the verification of understanding—along with the cycle of re-explanation and re-assessment when necessary—as the method’s core components [6,7].

Evidence regarding the effectiveness of Teach-Back has grown in recent years. A recent systematic review and meta-analysis of randomized controlled trials found that while the effects of Teach-Back on knowledge acquisition varied, improvements were reported in self-efficacy and certain health behaviors; however, the evidence was limited in certainty, and substantial heterogeneity was reported among the studies [8]. Similarly, a scoping review published in the *Healthcare* journal, which examined adults with cardiovascular disease, reported that Teach-Back could be beneficial in areas such as knowledge and understanding, self-care, and treatment adherence. In contrast, findings regarding readmission, healthcare utilization, and other clinical outcomes were more variable [9]. Taken together, these findings indicate that the effects of Teach-Back may differ according to target population, clinical context, delivery method, and outcomes assessed.

The broader evidence base in pediatric health literacy also supports the importance of communication interventions aimed at children and families. A recent scoping review of nursing interventions showed that strategies to improve health literacy among children and adolescents include education, communication, and individualized support [10]. Nevertheless, in pediatric health literacy, evidence remains needed on communication strategies that directly verify families’ understanding and application of health information [3].

Although findings from Teach-Back studies involving pediatric caregivers are promising, they span diverse clinical areas. A randomized controlled trial in a pediatric emergency department showed that a brief educational intervention—combining plain language, simplified written materials, a standard dosing syringe, and the “Teach-Back” method—improved parents’ and caregivers’ ability to accurately determine safe liquid medication doses compared with standard care [11]. A separate randomized clinical trial among caregivers of hospitalized children found that a health literacy-sensitive discharge intervention—incorporating pictogram-based medication instructions, dosing demonstrations, and structured “Teach-Back” and “Show-Back” components—reduced medication dosing errors at home and improved caregivers’ medication knowledge [5]. A recent systematic review on pediatric medication safety also supports the potential benefits of structured, multicomponent caregiver education in this area [4].

However, existing evidence syntheses primarily focus on adult populations, specific adult disease groups, or broader health literacy and medication safety interventions in the pediatric setting [3,4,8,9,10]. Randomized evidence on Teach-Back-based interventions for parents and primary caregivers across different pediatric clinical contexts therefore requires a focused synthesis. This systematic review evaluates the effectiveness of such Teach-Back-based interventions for parents and primary caregivers in pediatric healthcare, based on randomized controlled trials. In this context, we examined caregivers’ knowledge and understanding, caregiving and self-management skills, medication and care safety, self-efficacy, treatment adherence, healthcare utilization, caregiver-related outcomes, and child health outcomes.

## 2. Materials and Methods

### 2.1. Study Design and Protocol Registration

PRISMA 2020 provided the framework for reporting this systematic review [12]. The literature-search component was reported with reference to PRISMA-S (Preferred Reporting Items for Systematic Reviews and Meta-Analyses Literature Search Extension) to promote transparency and reproducibility [13]. The review protocol was registered in the International Prospective Register of Systematic Reviews (PROSPERO; CRD420261504251) before data extraction commenced. No amendments were made to the registered protocol.

### 2.2. Eligibility Criteria

Eligibility was assessed using prespecified criteria covering population, intervention, comparator, and study design. For the purposes of this review, the term “primary caregiver” referred to an individual primarily responsible for the child’s day-to-day health-related care, including parents, legal guardians, and other primary caregivers. Eligibility was determined by the individual’s caregiving role rather than their familial relationship or professional status. Therefore, professional caregivers were not excluded solely on the basis of their occupational status, provided they fulfilled the primary caregiving role and received the Teach-Back-based intervention.

Population: Eligible participants were parents, legal guardians, or primary caregivers of children or adolescents aged 0–18 years. We included parent–child or caregiver–child dyads in which the Teach-Back intervention was administered to the caregiver alone or with the child. The scope included caregivers of children with acute or chronic illnesses, as well as those utilizing preventive, routine, acute, or post-discharge pediatric healthcare services. We applied no restrictions on pediatric diagnosis, country, healthcare setting, or caregiver gender. Studies involving mixed age groups were deemed eligible only if data concerning pediatric caregivers could be analyzed separately.

We excluded studies in which the Teach-Back intervention was delivered solely to the child or adolescent without parent or caregiver participation; interventions aimed exclusively at healthcare professionals; studies focusing on caregivers of adult patients; and mixed-population studies in which pediatric caregiver data could not be obtained separately. We excluded studies primarily aimed at evaluating maternal, antenatal, or postpartum health outcomes. However, studies primarily targeting care for a newborn or child by a parent or primary caregiver were evaluated against pediatric eligibility criteria.

Intervention: Educational or communication interventions based on the “Teach-Back” method aimed at parents or primary caregivers were considered eligible. In this review, Teach-Back is defined as a closed-loop communication method in which the caregiver restates health information in their own words or demonstrates a taught skill; if gaps or misunderstandings are identified, the information is re-explained and understanding is reassessed. Teach-Back could be implemented either as a standalone intervention or as a clearly identifiable, core component of multicomponent interventions that included written materials, pictograms, demonstrations, “Show-Back,” plain language, digital tools, or telehealth support.

General communication training that merely recommended Teach-Back to healthcare professionals was not considered sufficient; the closed-loop verification process applied to the caregiver had to be a mandatory or clearly identifiable part of the intervention. We excluded one-way educational sessions that did not incorporate Teach-Back or an equivalent closed-loop verification process. Furthermore, studies in which Teach-Back was implemented identically in both study arms—meaning the randomized difference lay in another intervention component—were excluded. Studies were also excluded when the randomized contrast included an additional pharmacological, nutritional, procedural, or other clinical therapeutic co-intervention not provided to the comparison group, such that the contrast primarily reflected a non-educational treatment rather than the Teach-Back-based communication intervention. When the Teach-Back component was not fully described in the main trial report, intervention eligibility was verified using available study protocols, Supplementary Materials, or companion reports.

Comparator: Routine care, standard education, routine discharge education, attention-control conditions, or alternative educational interventions that did not include Teach-Back or equivalent closed-loop verification were considered appropriate comparators. Written, verbal, digital, demonstration-based, and multimedia educational approaches were also acceptable, as long as they did not use Teach-Back or another equivalent form of closed-loop verification.

Study design: Eligibility was restricted to randomized controlled trials. Individually randomized and cluster-randomized trials were included, together with pilot or feasibility RCTs when sufficient methodological and outcome information was available. Non-randomized controlled studies, quasi-experimental or quasi-randomized designs, single-group pre-test/post-test studies, observational and qualitative studies, case reports or case series, quality improvement projects, and reviews were excluded. Protocol-only publications and ongoing trial registrations were not included in the efficacy synthesis but were followed separately.

Study eligibility was not influenced by whether reported findings were favorable or statistically significant. The absence of one predefined outcome domain alone did not lead to exclusion; decisions were based on the prespecified population, intervention, comparator, and study-design criteria.

### 2.3. Information Sources and Search Strategy

Five bibliographic databases were searched: PubMed/MEDLINE, the Cochrane Central Register of Controlled Trials (CENTRAL), CINAHL Ultimate (EBSCOhost), Web of Science Core Collection, and Scopus. We additionally searched ClinicalTrials.gov and the World Health Organization International Clinical Trials Registry Platform (WHO ICTRP) to identify relevant ongoing trials, completed but unpublished studies, and studies for which a full report was not yet available.

**Figure 1:**
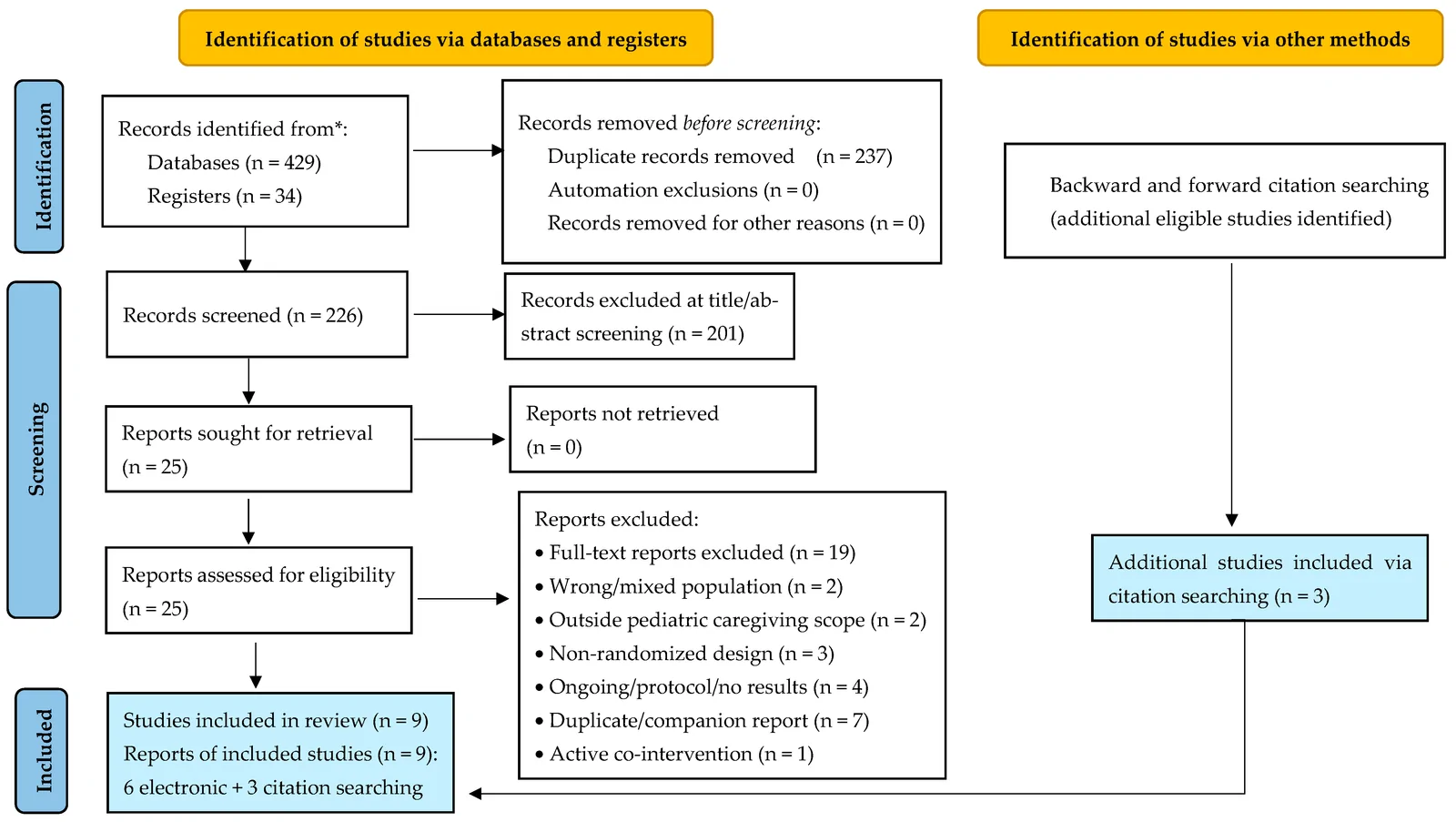
PRISMA 2020 flow diagram of the study selection process. Adapted from Ref. [12] under the CC BY 4.0 license. The flow diagram reflects the main searches completed by 16 September 2026 and the additional studies identified through citation searching. Supplementary trial registry searches conducted on 14 October 2026 identified no further eligible randomized controlled trials and are reported separately in Supplementary Table S1. The asterisk (*) indicates that database- and registry-specific record yields are reported in Supplementary Table S1.

The literature search was initiated on 10 August 2026, using PubMed/MEDLINE, CENTRAL, Web of Science Core Collection, Scopus, and CINAHL Ultimate. To ensure that the review remained current prior to finalization, update searches were conducted, with the final searches completed on 15 September 2026, for PubMed/MEDLINE, CENTRAL, Web of Science Core Collection, and Scopus, and on 16 September 2026, for CINAHL Ultimate. ClinicalTrials.gov and the WHO International Clinical Trials Registry Platform (ICTRP) were initially searched on 15 September and 16 September 2026, respectively. During manuscript revision, supplementary searches of ClinicalTrials.gov and the WHO International Clinical Trials Registry Platform (ICTRP) were conducted on 14 October 2026 using broader Teach-Back terminology without mandatory caregiver or pediatric search blocks. These searches retrieved 158 and 173 records, respectively, with 45 records sharing identical NCT identifiers across the two exports. After screening the supplementary registry records against the predefined eligibility criteria, no additional eligible randomized controlled trials were identified. These supplementary searches are reported separately in Supplementary Table S1 and were not included in the original search yield of 463 records presented in Figure 1. Consequently, the final number of included studies remained unchanged at nine randomized controlled trials.

Electronic searches were performed without restrictions on publication year or language, and no randomized controlled trial filter was applied. The search strategy was structured around three core conceptual blocks: (1) Teach-Back and equivalent closed-loop communication approaches, (2) parents/caregivers, and (3) the pediatric population. For PubMed/MEDLINE, Medical Subject Headings (MeSH) were combined with free-text terms. The same conceptual framework was adapted for the other databases using their respective controlled vocabulary, field tags, and database-specific syntax.

The Teach-Back conceptual block included terms such as “Teach-Back Communication,” “teach-back,” “teach back,” “teachback,” “show-back,” “show back,” “teach-to-goal,” “teach to goal,” “repeat-back,” “repeat back,” “closed-loop communication,” “closed loop communication,” and “return demonstration.” In the PubMed/MEDLINE strategy reported in Supplementary Table S1, the concepts represented by “tell-me-back”/“tell me back” and “demonstration-back”/“demonstration back” were searched using the broader Boolean expressions (tell[Title/Abstract] AND back[Title/Abstract]) and (demonstration[Title/Abstract] AND back[Title/Abstract]), respectively. These expressions differ from exact phrase searches and may retrieve additional records. The corresponding phrase variants were used in the strategies reported for the other bibliographic databases, as detailed in Supplementary Table S1. Terms used in the caregiver block included parent*, caregiver*, carer*, mother*, father*, guardian*, and famil*; terms used in the pediatric block included child*, pediatric*, paediatric*, infant*, newborn*, neonat*, toddler*, preschool*, adolescen*, and teen*. The asterisk (*) indicates truncation to capture variations of search terms.

We used broad terms to increase sensitivity; we deemed records eligible only if the intervention met the closed-loop Teach-Back structure defined in this review. Supplementary Table S1 presents the database- and registry-specific search strategies, final search dates, and record yields.

Beyond the electronic searches, the reference lists of included studies and relevant systematic reviews were examined for additional eligible records (backward citation tracking), and forward citation tracking was also conducted for the included studies. Records identified through these citation searches were assessed according to the same eligibility criteria used for the electronic database searches.

### 2.4. Selection of Studies

After combining the search results, duplicate records were identified and removed using Rayyan, with automated duplicate detection and resolution supplemented by manual verification of potentially duplicate or ambiguous records. Bibliographic identifiers (DOI and PMID), normalized titles, and other available bibliographic information were examined when necessary to resolve uncertain matches. Of the 463 records retrieved from bibliographic databases and trial registries, 237 duplicate records (51.2%) were removed, leaving 226 unique records for title and abstract screening. The remaining records were then independently evaluated by three researchers.

In the first stage, the three researchers independently screened titles and abstracts without seeing each other’s decisions. Records clearly meeting exclusion criteria were eliminated; the full texts of records deemed potentially eligible—or those for which eligibility could not be established with certainty from the title and abstract—were independently evaluated by the same three researchers in the second stage. Following the independent title/abstract and full-text screening stages, the three researchers verbally compared their eligibility decisions. No disagreements were identified during these comparisons, and all final eligibility decisions were concordant. However, individual reviewer-level screening decisions were not retained in a structured dataset suitable for retrospective statistical analysis. Therefore, a formal inter-rater agreement coefficient was not calculated for the study selection process.

When multiple articles, protocols, conference abstracts, or research registrations belonged to the same study, these records were linked and not counted as separate studies. We documented the main reasons for exclusion at the full-text stage, and the overall study-selection pathway is shown in the PRISMA 2020 flow diagram [12].

### 2.5. Data Extraction

Three researchers independently extracted data using a standardized form developed a priori. After independent data extraction, the researchers compared their data; they resolved discrepancies through discussion and consensus—including re-examining the original study reports—and agreed on the final dataset. When more than one report referred to the same study, information from the relevant reports was combined at the study level.

For each included study, we recorded author and publication year, country, healthcare setting, study design, unit of randomization, numbers of participants randomized and analyzed, caregiver characteristics, children’s age and clinical characteristics, the content and delivery of the Teach-Back intervention, the person or healthcare professional delivering the intervention, intervention duration and intensity, supportive educational components, comparator intervention, and follow-up duration. We also extracted the outcomes assessed, outcome definitions and measurement tools, assessment time points, numerical outcome data by group, and reported effect estimates. We also recorded study registration numbers and protocol details, funding sources, and reported conflicts of interest where applicable.

### 2.6. Outcome Measures

Predefined primary outcome domains were evaluated in two groups: (1) the parent’s or caregiver’s level of knowledge and understanding regarding health information, and (2) performance in accurately and safely carrying out the care task targeted by the Teach-Back intervention. Knowledge and understanding outcomes included measures of medication or treatment information, accurate verbalization of safe dosage information, and comprehension of care instructions. Care performance outcomes included accurate medication dosing, the correct execution of the taught care procedure, and other observed or reported safe care skills.

Secondary outcome domains encompassed caregiver self-efficacy, treatment or medication adherence, caregiver burden, and other caregiver-related outcomes; healthcare utilization; disease or symptom control in the child; clinical health outcomes; relevant physiological measurements; and other caregiving or self-management outcomes.

We recorded all relevant post-intervention assessment time points reported by the studies; we evaluated follow-up periods that differed to a clinically significant extent separately during synthesis. Eligibility did not depend on whether a particular outcome domain was reported, on the direction of the observed effect, or on whether the finding reached statistical significance.

### 2.7. Risk of Bias Assessment

Risk of bias in the included randomized controlled trials was evaluated using the Cochrane Risk of Bias 2 (RoB 2) tool [14,15]. In this review, the term “principal outcomes” refers to the outcome-specific results selected to inform the narrative synthesis and the corresponding outcome-domain GRADE assessments, rather than necessarily the primary outcomes designated by the original trial investigators. Outcome selection was guided by the predefined outcome domains described in Section 2.6, with attention to clinical relevance and the results contributing to the outcome-domain summaries. Statistical significance or the direction of the observed effect was not used as a selection criterion. Because risk of bias may differ according to outcome measurement, missing outcome data, and selective reporting, separate outcome-specific assessments were considered necessary when a trial contributed results to more than one clinically distinct outcome domain. In particular, caregiver medication knowledge was distinguished from observed medication dosing accuracy, and diabetes self-care was distinguished from HbA1c. The assessed study–outcome pairs and their domain-level justifications are presented in Table 1 and Supplementary Table S2, respectively.

For the purposes of the RoB 2 assessment, principal outcomes were defined as the results contributing directly to the clinically important outcome domains summarized in Table 2. Outcome selection was guided by the prespecified outcome domains described in Section 2.6, the clinical relevance of the result, the measurement approach, and the relevant follow-up time point, rather than the statistical significance or direction of the findings. When multiple critical outcomes from the same trial informed the evidence synthesis, these were treated as distinct assessment targets because the risks associated with missing outcome data, outcome measurement, and selective reporting may differ between results.

For each assessed outcome, five RoB 2 domains were considered: (1) bias arising from the randomization process, (2) bias due to deviations from intended interventions, (3) bias due to missing outcome data, (4) bias in measurement of the outcome, and (5) bias in selection of the reported result. Domain-level and overall judgments were categorized as “low risk,” “some concerns,” or “high risk” [14,15].

Three researchers independently completed outcome-specific RoB 2 assessments for 14 results from nine randomized controlled trials. Inter-rater agreement was evaluated using Fleiss’ kappa based on the three researchers’ individual judgments before consensus. Complete agreement was observed for all 14 overall RoB 2 judgments (100%; Fleiss’ κ = 1.000). At the domain level, complete agreement among all three researchers was achieved for 64 of 70 outcome-domain assessments (91.4%). Domain-specific Fleiss’ kappa values were 0.810 for D1, 0.560 for D2, 1.000 for D3, 0.843 for D4, and 1.000 for D5. Six domain-level disagreements were identified in the independent assessments and were subsequently resolved through discussion and consensus. No disagreements were identified in the overall RoB 2 judgments. Detailed domain-level justifications supporting the final consensus judgments are provided in Supplementary Table S2.

### 2.8. Data Synthesis and Statistical Analysis

Study characteristics, intervention features, and outcomes were presented in tables and integrated through a structured narrative synthesis. Studies were organized according to outcome domain and clinical context, and were compared by considering intervention characteristics, comparators, measurement methods, and follow-up times. Effect measures were reported as presented in the included studies, including risk ratios (RRs), odds ratios (ORs), mean differences, absolute differences, and corresponding 95% confidence intervals where available.

The synthesis of findings did not rely on a simple vote-counting approach based solely on statistical significance; instead, we evaluated reported effect estimates, group-level numerical results together with the direction and magnitude of effects and the methodological characteristics of the evidence.

Meta-analysis was not undertaken because no outcome domain contained at least two studies that were sufficiently comparable in terms of outcome definition, measurement approach, intervention structure, comparator, and follow-up time to yield a clinically meaningful pooled estimate. Even within the same broad outcome domains, studies used different definitions and methods of assessment, and the Teach-Back component was often embedded within different multicomponent interventions. For example, even within the medication-safety domain, studies differed in the definition of dosing error, assessment procedures, intervention components, and timing of outcome measurement. Pooling such studies was therefore considered likely to produce a summary estimate with limited clinical interpretability. Accordingly, findings were integrated using a structured narrative approach, considering the outcome domain, clinical context, intervention characteristics, and follow-up duration.

Because meta-analysis was not performed, reporting of the narrative synthesis was informed by the Synthesis Without Meta-analysis (SWiM) guideline [16].

### 2.9. Reporting Bias and Small-Study Effects

When trial registrations or published protocols were available, we checked them against the corresponding final reports to identify possible differences in the outcomes reported. Trial registries were also examined for relevant ongoing studies and completed studies whose findings had not yet been published.

Small-study effects were not formally evaluated. Neither funnel plot asymmetry nor Egger’s regression test was assessed because the review did not include a meta-analysis and too few studies contributed to individual outcome domains to support a reliable assessment [15,17].

### 2.10. Assessment of Certainty of Evidence

Certainty of evidence was evaluated for the key outcome domains using the Grading of Recommendations Assessment, Development and Evaluation (GRADE) approach [18]. Evidence derived from randomized controlled trials started at high certainty and was downgraded when warranted for risk of bias, inconsistency, indirectness, imprecision, or publication bias [18]. Final certainty ratings were expressed as high, moderate, low, or very low.

As no meta-analysis was undertaken, the GRADE judgment for each outcome domain was based on the effect estimates described in the narrative synthesis, the direction and magnitude of the findings, the number and size of the contributing studies, and relevant methodological limitations.

**Table 1:** Risk of bias assessment of the included randomized controlled trials using RoB 2.

Study/Outcome	D1 *	D2	D3	D4	D5	Overall
Yin et al., 2008 [19]—observed dosing error	Some concerns	Low	Some concerns	Low	Some concerns	Some concerns
Yin et al., 2008 [19]—incorrect knowledge of daily dose medication frequency	Some concerns	Low	High	Some concerns	Some concerns	High
Chen et al., 2018 [20]—deviation from ideal toothpaste amount	Some concerns	Low	Low	Low	Some concerns	Some concerns
Ghoneim & Fathalla, 2018 [21]—maternal self-efficacy	Some concerns	Low	Some concerns	High	Some concerns	High
Li et al., 2020 [11]—safe dose knowledge at 48–72 h	Some concerns	Low	High	Some concerns	Low	High
Eakin et al., 2020 [22]—TRACK asthma control	Low	Low	Some concerns	Some concerns	Some concerns	Some concerns
Eakin et al., 2020 [22]—Asthma-related hospitalization	Low	Low	Some concerns	Some concerns	Some concerns	Some concerns
Carroll et al., 2024 [5]—correct caregiver-reported medication dose	Low	Low	Some concerns	Some concerns	Some concerns	Some concerns
Carroll et al., 2024 [5]—observed home dosing error	Low	Low	Some concerns	Low	Low	Some concerns
Frey et al., 2025 [23]—symptom-free days at 6 months	Low	Low	High	Some concerns	Low	High
Frey et al., 2025 [23]—Asthma-related acute healthcare use	Low	Low	Low	Low	Some concerns	Some concerns
Schade et al., 2026 [24]—7-day visible plaque index	Low	Some concerns	Some concerns	Low	Some concerns	Some concerns
Zelhagh et al., 2026 [25]—SDSCA self-care	Low	Low	Low	High	Some concerns	High
Zelhagh et al., 2026 [25]—HbA1c	Low	Low	Low	Low	Some concerns	Some concerns

* D1, bias arising from the randomization process; D2, bias due to deviations from intended interventions; D3, bias due to missing outcome data; D4, bias in measurement of the outcome; D5, bias in selection of the reported result. Green = low risk; yellow = some concerns; red = high risk. RoB 2 judgments are outcome-specific. Separate outcome-specific RoB 2 assessments were conducted for Yin et al. [19] (observed dosing error and medication knowledge), Carroll et al. [5] (observed home dosing error and medication knowledge), Eakin et al. [22] (TRACK asthma control and asthma-related hospitalization), Frey et al. [23] (symptom-free days and asthma-related acute healthcare use), and Zelhagh et al. [25] (SDSCA self-care and HbA1c).

**Table 2:** Summary of Findings and GRADE assessment of the certainty of evidence.

Outcome	Studies/Participants	Narrative Effect Summary	GRADE Certainty	Main Reason(s) for Downgrading
Medication dosing accuracy/safety [5,19]	2 RCTs; 443 randomized; 332 contributed direct observed dosing outcomes	Both trials favored Teach-Back-based interventions. Yin et al. [19] reported markedly fewer observed dosing errors; Carroll et al. [5] reported any dosing error in 30.4% vs. 54.2% and mean percentage dosing error of 1.0% vs. 3.3%.	MODERATE	Serious risk of bias
Caregiver medication/safe-dose knowledge [5,11,19]	3 RCTs; 592 randomized; analyzable n varied by outcome	Several medication-related knowledge outcomes favored Teach-Back-based interventions, although benefits were not consistent across all knowledge items. Li et al. [11] reported correct safe-dose knowledge in 71% versus 45% of caregivers (RR 1.58, 95% CI 1.12–2.24), but follow-up was incomplete and differed between groups (35/66 versus 62/83). Yin et al. [19] and Carroll et al. [5] reported improvements in selected medication knowledge outcomes, while other knowledge measures showed no clear between-group differences.	LOW	Serious risk of bias (−1): substantial and differential loss to follow-up in Li et al. [11], together with outcome-specific methodological concerns in Yin et al. [19] and Carroll et al. [5]. Serious imprecision (−1): limited analyzable sample sizes for individual medication-knowledge outcomes, a wide confidence interval for the effect reported by Li et al., and uncertainty regarding the magnitude of clinically important benefits.
Oral health/care performance [20,24]	2 RCTs; 163 participants	No consistent overall superiority was demonstrated. Chen et al. [20] found no significant added benefit over visual guidance (*p* = 0.40); Schade et al. [24] reported benefit for visible plaque only in a psychosocial subgroup.	VERY LOW	Serious risk of bias, inconsistency, and imprecision
Asthma control (TRACK) [22]	1 RCT; 398 randomized	At 3 months, Asthma Basic Care plus Head Start education improved TRACK scores compared with Head Start education alone (β = 6.26; 95% CI: 1.77–10.75).	LOW	Serious risk of bias and imprecision; the confidence interval was wide (1.77–10.75 points). A change of ≥10 TRACK points is considered clinically meaningful for individual patients, although this individual-level threshold does not directly establish a clinically important between-group effect.
Asthma-related healthcare utilization [22,23]	2 RCTs; 424 randomized	Eakin et al. [22] reported fewer oral corticosteroid courses and lower hospitalization odds (OR 0.36; 95% CI 0.21–0.61). The small TEACHH pilot did not show a clear reduction in acute healthcare use.	VERY LOW	Serious risk of bias, inconsistency, and imprecision
Diabetes self-care (SDSCA) [25]	1 RCT; 60 participants	Self-care improved substantially with Teach-Back versus routine education (post-intervention difference vs. control ≈ 24.9 points; *p* < 0.001), but Teach-Back was not clearly superior to multimedia education.	VERY LOW	Very serious risk of bias and serious imprecision
HbA1c [25]	1 three-arm RCT; 60 randomized participants (20 per group); 40 participants in the Teach-Back versus routine education comparison.	HbA1c decreased from 8.53 ± 0.77 to 5.60 ± 0.42 with Teach-Back versus 8.17 ± 0.71 to 7.84 ± 0.61 with routine care; Teach-Back was not clearly superior to multimedia education.	LOW	Serious risk of bias (concerns regarding prespecification and selection of the reported HbA1c result) and serious imprecision (evidence from a single small trial, with 20 participants per group). The unusually large HbA1c reductions in both active education groups warrant additional caution when interpreting the findings.
Caregiver self-efficacy [21]	1 RCT; 60 participants	One RCT reported improved maternal self-efficacy after Teach-Back-based discharge education compared with routine discharge education.	VERY LOW	Very serious risk of bias and serious imprecision

Evidence from randomized trials started at high certainty. Certainty was downgraded for serious or very serious concerns in risk of bias, inconsistency, indirectness, imprecision, or publication bias. Because fewer than 10 studies contributed to every outcome, publication bias could not be formally assessed; no outcome was downgraded solely for inability to perform a formal test. For outcomes informed by a single study, inconsistency could not be assessed and was therefore not used as a basis for downgrading. Certainty ratings refer to the Teach-Back-based interventions as delivered in the included trials and should not be interpreted as certainty regarding the isolated effect of Teach-Back.

## 3. Results

### 3.1. Study Selection

We identified 463 records through database and trial registry searches, including 429 records from bibliographic databases and 34 records from trial registries. After removing 237 duplicate records, 226 unique records remained for title and abstract screening. Of these, 201 records were excluded, and 25 reports were assessed for full-text eligibility. Nineteen reports were excluded at the full-text stage for the reasons shown in the PRISMA 2020 flow diagram, leaving six randomized controlled trials identified through database and registry searches. Backward and forward citation searching identified three additional eligible randomized controlled trials. Therefore, nine randomized controlled trials were included in the final review.

The number of records retained or excluded at each stage of the selection process is presented in the PRISMA 2020 flow diagram (Figure 1) [12].

### 3.2. Characteristics of Included Studies

The nine randomized controlled trials were published over the period from 2008 to 2026 [5,11,19,20,21,22,23,24,25]. Across these trials, 1299 participants were randomized, and individual study samples ranged from 26 to 398 participants. Of the nine studies, six were conducted in the United States [5,11,19,20,22,23], while Egypt [21], Brazil [24], and Iran [25] each contributed one study.

The clinical contexts of the studies varied considerably. Three studies evaluated pediatric medication safety and dosing accuracy [5,11,19], while two focused on oral health practices [20,24]. Other studies addressed caregiver education for congenital heart disease [21], childhood asthma management [22,23], and self-care education for type 1 diabetes [25]. While some studies implemented Teach-Back as a direct educational approach, others used it as a core component of multicomponent interventions that included additional elements such as pictograms, plain language, demonstration, Show-Back, written materials, or telehealth support.

Follow-up assessments ranged from immediately after the educational intervention to 12 months after the intervention. The main characteristics of the nine included trials are provided in Table 3. A component-level comparison of the Teach-Back interventions, accompanying educational strategies, comparator conditions, and available information on the equivalence of educational attention is presented in Supplementary Table S3. This comparison highlights that most trials evaluated multicomponent educational packages incorporating Teach-Back rather than the isolated effect of Teach-Back itself. Additional details on intervention delivery, educator training, session duration, Teach-Back repetitions, and the assessment or monitoring of intervention fidelity are summarized in Supplementary Table S4.

### 3.3. Findings Regarding Intervention Effectiveness

#### 3.3.1. Medication Dosing Safety, Medication Knowledge, and Adherence

Researchers evaluated medication safety and dosing outcomes in three randomized controlled trials involving 592 caregivers [5,11,19]. In all three studies, the Teach-Back method was implemented alongside additional health literacy components, such as plain language, written or pictographic materials, dosing devices and/or demonstration-based instruction. Nevertheless, the trials varied in how outcomes were defined and measured and in the timing of follow-up assessments.

In Yin et al. [19], observed liquid-medication dosing errors were substantially less frequent among caregivers receiving the HELPix intervention. Using a deviation of more than 20% from the recommended dose as the definition of a dosing error, 5.4% of caregivers in the intervention group and 47.8% in the control group made errors with daily medications, corresponding to an absolute risk reduction of 42.4% (95% CI: 24.0–57.0%). For as-needed medications, dosing errors occurred in 15.6% of the intervention group and 40.0% of the control group, with an absolute risk reduction of 24.4% (95% CI: 8.7–38.8%). Among caregivers giving daily medications, non-adherence occurred in 9.3% of the intervention group versus 38.0% of the control group. Caregivers receiving the intervention also showed better knowledge of medication preparation and the appropriate dosing frequency for daily medications [19].

In Li et al. [11], 48–72 h after discharge from a pediatric emergency department, 71% (25/35) of parents or guardians receiving the Teach-Back-based intervention correctly identified a safe dose of liquid acetaminophen or ibuprofen, compared with 45% (28/62) receiving standard care. Correct identification of the safe dose was more frequent in the intervention group (RR = 1.58; 95% CI: 1.12–2.24). The association remained after adjustment for caregiver health literacy and language (adjusted RR = 1.50; 95% CI: 1.06–2.13) [11].

Carroll et al. [5] studied caregivers of children discharged with a new prescription for a liquid medication. At home, the mean percentage dosing error was 1.0% following the intervention and 3.3% following standard counseling (absolute difference 2.3%; 95% CI: 1.0–3.6%; *p* < 0.001). Any dosing error occurred in 30.4% (24/79) of caregivers in the intervention group compared with 54.2% (39/72) in the control group (*p* = 0.003). Caregivers in the intervention group were also more likely to correctly report the medication dose (93.4% vs. 79.7%; *p* = 0.03), treatment duration (85.5% vs. 71.0%; *p* = 0.04), and at least two medication adverse effects (78.9% vs. 18.8%; *p* < 0.001). In contrast, the groups did not differ significantly in drug name, indication, frequency of administration, or storage information [5].

Overall, all three studies demonstrated a trend favoring interventions that included the Teach-Back method [5,11,19]. However, the results were not quantitatively pooled because of the multicomponent nature of the interventions, variations in how dosing safety and medication knowledge were defined and assessed, and inconsistencies in evaluation timing; instead, they were analyzed through a structured narrative synthesis.

#### 3.3.2. Oral Health and Care Skills

Oral health-related outcomes were evaluated in two randomized controlled trials [20,24]. Chen et al. [20] examined the effect of adding the Teach-Back method to visual aid counseling on improving the application of age-appropriate amounts of fluoride toothpaste among 85 caregivers of children aged 1–6 years. Across all participants, deviation from the ideal toothpaste amount decreased after using visual aids (*p* = 0.008). Although caregivers in the Teach-Back group applied amounts closer to the ideal, the difference in mean deviation between the Teach-Back group and the visual aid-only group was not statistically significant (*p* = 0.40) [20].

Schade et al. [24] assigned 78 preschool children and their caregivers from a rural setting to oral hygiene education delivered either through standard guidance or using the Teach-Back method. Seven days after the intervention, oral hygiene was assessed with the Visible Plaque Index (VPI) and the Simplified Oral Hygiene Index (OHI-S). Within the subgroup characterized by a low sense of coherence and a higher internal parental locus of control, participants receiving Teach-Back had fewer teeth with visible plaque. By contrast, reductions in OHI-S scores were observed irrespective of caregiver psychosocial characteristics or the educational approach used [24].

Overall, while these two studies suggest that Teach-Back may offer potential benefits in oral health-related care practices, they did not demonstrate consistent superiority over standard or visual guidance [20,24]. Due to differences in intervention modalities, outcomes assessed, and follow-up periods, the studies were not quantitatively pooled; instead, the findings were evaluated through a structured narrative synthesis.

#### 3.3.3. Caregiver Self-Efficacy and Discharge Education

Caregiver self-efficacy was evaluated in a randomized controlled trial. Ghoneim and Fathalla [21] randomized 60 children with congenital heart disease and their mothers to either Teach-Back-based discharge education or routine discharge education. Following the intervention, mothers in the experimental group had higher self-efficacy than those in the control group, with improvement observed among mothers who received Teach-Back-based education. The intervention group also reported high levels of satisfaction [21].

Because the available outcome data did not provide a standardized effect size or confidence interval, these findings were not quantitatively pooled and were instead evaluated within a structured narrative synthesis.

#### 3.3.4. Asthma Control, Symptoms, and Healthcare Utilization

In pediatric asthma, two randomized controlled trials evaluated Teach-Back or equivalent closed-loop verification methods as part of multicomponent family education [22,23].

Eakin et al. [22] studied 398 preschool children with asthma and their primary caregivers attending Head Start programs. Compared with Head Start asthma education alone, the combined Asthma Basic Care family education and Head Start education produced a greater improvement in asthma control over 12 months (β = 6.26; 95% CI: 1.77–10.75; *p* < 0.001). The intervention group also had fewer courses of oral corticosteroids (β = −0.61; 95% CI: −1.13 to −0.09; *p* = 0.02) and lower odds of hospitalization (OR = 0.36; 95% CI: 0.21–0.61; *p* < 0.001) [22]. However, because the intervention included multiple components at the home and school levels, we cannot attribute the observed effect solely to the Teach-Back component.

In the TEACHH pilot RCT conducted by Frey et al. [23], 26 child–caregiver pairs were randomized to either a program comprising Teach-Back/Show-Back, visual materials, color- and shape-coded medication labels, and two telehealth follow-up sessions, or standard care. All in-hospital education sessions were completed, and 77% of the planned virtual follow-up visits were carried out. Both groups reported improvements in symptoms and caregiver quality of life over time; however, due to the small sample size, a reliable comparison of efficacy between groups regarding clinical outcomes could not be performed [23].

In general, while asthma studies indicate that multicomponent educational approaches incorporating Teach-Back show potential benefits for asthma control and certain healthcare utilization outcomes, studies varied considerably in sample size, intervention intensity, components, and outcomes assessed. Therefore, the studies were not quantitatively pooled, and the findings were evaluated within a structured narrative synthesis.

#### 3.3.5. Diabetes Self-Care and Glycemic Control

In the trial by Zelhagh et al. [25], 60 children aged 6–12 years with type 1 diabetes and their mothers were randomly allocated to Teach-Back education, multimedia education, or routine education. Before the intervention, self-care scores were comparable across groups (*p* = 0.971), as were HbA1c levels (*p* = 0.149).

After the intervention, mean self-care scores rose from 34.50 ± 2.56 to 59.15 ± 3.40 in the Teach-Back group, while the control group had a post-intervention score of 34.25 ± 3.40. A significant between-group difference was observed (*p* < 0.001), with an unadjusted mean difference of approximately 24.9 points between the Teach-Back and control groups (*p* < 0.001). By comparison, Teach-Back and multimedia education did not differ significantly in self-care scores (mean difference = 1.6; *p* = 0.277) [25].

For glycemic control, HbA1c decreased from 8.53 ± 0.77 to 5.60 ± 0.42 in the Teach-Back group and from 8.54 ± 0.52 to 5.66 ± 0.37 in the multimedia education group. In the control group, HbA1c changed from 8.17 ± 0.71 to 7.84 ± 0.61. Differences among the groups after the intervention were significant (*p* < 0.001), and the post-hoc comparison of Teach-Back with routine education was also significant (*p* < 0.001) [25].

Although these HbA1c values were reproduced as reported in the original trial, the reductions of approximately 2.9 percentage points in both active education groups over the three-month follow-up were unusually large for brief educational interventions. Furthermore, the trial report provided insufficient detail on concurrent treatment changes or other factors potentially influencing glycemic control to fully explain the magnitude of these reductions. Therefore, these findings warrant cautious interpretation [25].

Overall, Teach-Back-based education was associated with better diabetes self-care and glycemic control than routine education in this trial. However, Teach-Back did not show clear superiority over multimedia education, and these findings are based on a single study.

#### 3.3.6. General Pattern of Results and Suitability for Quantitative Synthesis

Effects frequently favored Teach-Back-based interventions; however, the consistency, magnitude, and certainty of these effects varied substantially across outcome domains and clinical contexts. While the most consistent benefits were observed in outcomes related to medication dosing safety and caregivers’ medication knowledge [5,11,19], outcomes related to oral health and asthma appeared more context-dependent and associated with multicomponent interventions [20,22,23,24]. Only single studies evaluated outcomes related to caregiver self-efficacy, diabetes self-care, and glycemic control [21,25].

Considerable variation was observed across studies in intervention content, comparator conditions, outcome definitions, measurement approaches, and length of follow-up. While some studies implemented Teach-Back as a direct educational approach, others used it alongside additional components—such as pictograms, demonstrations, Show-Back, telehealth support, school-based education, or multimedia.

No outcome domain contained two studies sufficiently similar to support a clinically meaningful pooled estimate, and meta-analysis was therefore not undertaken. Accordingly, the results were synthesized narratively according to outcome domain, clinical context, intervention characteristics, and follow-up duration.

### 3.4. Risk of Bias in Included Studies

Outcome-level risk of bias was evaluated for all nine randomized controlled trials using the Cochrane Risk of Bias 2 (RoB 2) tool. A total of 14 outcome-specific assessments were conducted across the nine trials, including separate assessments of medication knowledge outcomes for Yin et al. [19] and Carroll et al. [5], and asthma-related healthcare utilization outcomes for Eakin et al. [22] and Frey et al. [23]. None of these assessments was judged to be at low risk of bias; nine were judged as having some concerns and five as having a high risk of bias.

The issues most frequently contributing to risk of bias were missing outcome data, measuring self-reported outcomes in educational interventions where blinding was not possible, and concerns about the selection of reported outcomes. However, because RoB 2 assessments are outcome-specific, we reached different overall judgments for different outcomes within the same study. An important distinction was identified between observed medication dosing outcomes and caregiver-reported medication knowledge outcomes. In Yin et al. [19], the inability to maintain assessor blinding during follow-up raised concerns regarding the measurement of medication knowledge, although responses were evaluated using structured questions. In Carroll et al. [5], medication knowledge was assessed through a caregiver-completed follow-up survey, with incomplete outcome data and potential influences related to the use of educational materials during assessment. These methodological differences require outcome-specific consideration and should not be assumed to confer identical risks of bias across medication dosing and knowledge outcomes.

This was evident in Zelhagh et al. [25], for whom the self-reported SDSCA self-care outcome received an overall “high risk” judgment, whereas the objectively measured HbA1c outcome was judged as having “some concerns.” Table 1 presents domain-specific and overall RoB 2 assessments.

### 3.5. Certainty of Evidence

Certainty was evaluated for each outcome domain according to the GRADE framework. Evidence for medication dosing accuracy/safety was judged to be of moderate certainty, whereas evidence for caregiver medication or safe-dose knowledge was judged to be of low certainty. Asthma control and HbA1c were supported by low-certainty evidence, whereas evidence for oral health/care performance, asthma-related healthcare utilization, and diabetes self-care was rated as very low certainty.

For caregiver self-efficacy, evidence was available from only one small randomized controlled trial and was therefore rated as very low certainty because of very serious risk of bias and imprecision. Risk of bias and imprecision most often downgraded the certainty of evidence; inconsistency also limited certain outcomes, such as oral health/care performance and asthma-related healthcare utilization. Table 2 presents GRADE assessments and the primary reasons for downgrading, categorized by outcome domain. A detailed outcome-specific linkage between the RoB 2 judgments and GRADE downgrading decisions, including the rationale for certainty ratings, is provided in Supplementary Table S5.

## 4. Discussion

This review brought together evidence from nine randomized controlled trials examining Teach-Back-based interventions for parents and primary caregivers across pediatric healthcare settings. The most consistent positive findings were observed regarding medication dosing accuracy and safety, as well as caregivers’ knowledge of medication and safe dosing; however, the certainty of evidence was moderate for medication dosing accuracy/safety and low for caregiver medication/safe-dose knowledge. By contrast, certainty was low for asthma control and HbA1c and very low for oral health and care performance, asthma-related healthcare utilization, diabetes self-care, and caregiver self-efficacy. Furthermore, most studies implemented Teach-Back alongside additional components such as pictograms, plain language, demonstration, Show-Back, written materials, or telehealth support. Therefore, while current findings suggest that interventions incorporating Teach-Back may offer benefits—particularly for medication safety—the magnitude and generalizability of the overall intervention effects, as well as the independent contribution of Teach-Back, should be interpreted with caution.

Medication safety showed the most consistent pattern of benefit in the included evidence. In all studies conducted by Yin et al. [19], Li et al. [11], and Carroll et al. [5], interventions incorporating Teach-Back yielded more favorable outcomes than standard care regarding caregivers’ medication dosing accuracy and/or medication knowledge. These findings align with a recent systematic review of medication education interventions for caregivers of pediatric patients. Giva et al. [4] reported that multicomponent approaches—incorporating health literacy-sensitive education, structured counseling, and standard dosing tools—improved caregivers’ medication knowledge and reduced home medication dosing errors. However, in most studies in this review and in the broader literature, Teach-Back was implemented alongside plain language, pictograms, demonstrations, Show-Back, or standard dosing tools. Therefore, the observed benefit cannot be attributed solely to Teach-Back; rather, existing evidence suggests that health literacy-sensitive, multicomponent educational approaches incorporating Teach-Back can support pediatric medication safety.

In clinical areas beyond medication safety, findings are more variable and less certain. Studies on oral health failed to demonstrate a consistent superiority of Teach-Back over standard or visually guided instruction [20,24]. While a larger asthma study reported positive clinical outcomes, Teach-Back was only one component of a home- and school-based multicomponent program; meanwhile, the small sample size of the TEACHH pilot study limited definitive conclusions regarding clinical efficacy [22,23]. In the type 1 diabetes trial, both Teach-Back and multimedia education were associated with substantially lower HbA1c values than routine education at the three-month follow-up [25]. However, the reported reductions of approximately 2.9 percentage points in both intervention groups raise concerns about clinical plausibility, particularly given the relatively brief educational interventions and limited information on concomitant treatment changes or other potential influences on glycemic control. Although the reported values were verified against the original trial publication, their magnitude remains difficult to explain based on the available information. Moreover, the similarity of the HbA1c results in the Teach-Back and multimedia groups does not support a distinct advantage of Teach-Back for glycemic control. Accordingly, these findings should be considered preliminary and interpreted cautiously, particularly because they originate from a single small trial. Positive findings on caregiver self-efficacy also come from only a single, small study [21]. This pattern suggests that the impact of Teach-Back may vary by outcome domain, intervention content, and the clinical context in which it is applied.

An additional methodological consideration is that differences in the certainty and consistency of findings may partly reflect how outcomes were measured, rather than the clinical domain alone. More consistent favorable findings were observed for directly assessed medication dosing accuracy [5,19], for which the RoB 2 measurement-of-outcome domain (D4) was judged to be at low risk of bias. In contrast, caregiver-reported outcomes, particularly maternal self-efficacy [21] and diabetes self-care behaviors [25], were more susceptible to measurement bias because participants were aware of their intervention allocation; both outcomes received high-risk judgments for D4. Nevertheless, objective measurement does not necessarily imply a beneficial intervention effect or high certainty of evidence. For example, the objectively measured toothpaste quantity outcome did not demonstrate a clear additional benefit of Teach-Back [20], while the objectively assessed HbA1c findings [25] remained uncertain because of concerns about clinical plausibility, risk of bias, and imprecision. Thus, outcome measurement methods should be considered alongside clinical context, intervention characteristics, and other methodological limitations when interpreting Teach-Back-based interventions. Future trials should prioritize standardized and objectively assessed outcomes where feasible, blinded outcome assessment when possible, and transparent prespecification of outcomes and analyses.

A similar pattern has been described in the wider Teach-Back literature. A recent systematic review and meta-analysis involving adult patients and care partners did not identify a consistent effect on knowledge acquisition, although improvements were reported for self-efficacy and adherence to health behaviors. The certainty supporting these findings was low to very low, and substantial heterogeneity was present across studies [8].

Studies have also reported that the operational definition and implementation steps of Teach-Back vary considerably across studies [7]. Therefore, clearly defining the core components of Teach-Back, assessing intervention fidelity, and using comparable outcome measures in future pediatric research are crucial for strengthening the evidence base.

In this review, heterogeneity across studies extended beyond measurement tools and follow-up times to include intervention structure and targeted clinical outcomes. While some studies implemented Teach-Back as a standalone educational method, others combined it with additional components such as pictograms, plain language, demonstration, Show-Back, telehealth support, or school-based education. Similarly, even within the same general outcome domain, definitions of outcomes, assessment methods, and follow-up durations varied. Consequently, quantitatively pooling the studies—even if statistically feasible—would have yielded a pooled effect estimate that was difficult to interpret clinically. In light of this, we evaluated the findings through a narrative synthesis structured by outcome domain and clinical context. While this approach highlights how the intervention’s effect varies across different pediatric care settings, it limits the ability to express Teach-Back’s impact through a single, generalizable effect size.

Several methodological features strengthen this review. Restricting inclusion to randomized controlled trials allowed the review to focus on comparative evidence concerning the effectiveness of Teach-Back-based interventions. The literature search was comprehensive, covering five major bibliographic databases, ClinicalTrials.gov, and the WHO ICTRP, without limitations by publication year or language. Study selection, data extraction, and risk-of-bias assessment were conducted independently by the researchers. In addition, outcome-level assessment with RoB 2 and outcome-domain-level evaluation with GRADE allowed the findings to be interpreted in relation to both statistical results and the methodological credibility of the underlying evidence.

Several limitations should also be considered when interpreting these findings. The review included only nine studies, and many outcome domains were assessed by only one or two studies. Considerable variation in clinical context, intervention content, comparator conditions, outcome definitions, and follow-up duration prevented meta-analysis and limited the possibility of deriving a single common effect estimate. In several included trials, Teach-Back was combined with other educational components, including pictograms, plain-language materials, dosing tools, demonstrations, Show-Back, or telehealth support [5,11,19,22,23]. Because these components were delivered as part of integrated intervention packages rather than independently randomized, the observed effects represent the combined educational interventions and cannot be attributed exclusively to Teach-Back. In contrast, Chen et al. [20] compared visual-aid counseling with and without Teach-Back, providing a more direct assessment of its additional contribution; however, no statistically significant between-group difference in mean deviation from the recommended toothpaste amount was observed. Therefore, differences in intervention components and comparator conditions limit the ability to isolate the independent effect of Teach-Back and should be considered when interpreting the findings across pediatric healthcare settings. None of the principal-outcome assessments was judged to be at low risk of bias, and concerns related particularly to missing outcome data, self-reported measures when blinding was not feasible, and selection of the reported result contributed to lower certainty of evidence. Finally, six of the nine studies were conducted in the United States, and several clinical areas were represented by only a single study, which may restrict the applicability of the findings across different healthcare systems, sociocultural settings, and pediatric populations.

Notably, three of the nine included randomized controlled trials (33.3%) were identified through backward or forward citation tracking rather than the original database and trial registry searches. This finding suggests that electronic searches alone may not fully capture the available evidence on Teach-Back-based interventions in pediatric healthcare. Possible explanations include inconsistent indexing, variation in terminology used to describe Teach-Back, and the incorporation of Teach-Back within broader multicomponent educational interventions. Although the specific reasons these studies were not retrieved electronically could not be established, this finding highlights the importance of combining comprehensive electronic searches with systematic citation tracking. Future reviews and updates should retain backward and forward citation searching and consider additional terminology identified from relevant studies to improve retrieval sensitivity.

Current findings suggest that Teach-Back may be a clinically useful communication component, particularly in situations—such as medication management—where accurate understanding and correct implementation are directly linked to patient safety. However, since Teach-Back is often implemented as part of multicomponent interventions, it remains unclear to what extent specific components contribute to the outcomes. Future studies should clearly and consistently define the core implementation steps of Teach-Back, assess intervention fidelity, and use comparison groups that isolate Teach-Back’s contribution from other educational components. Furthermore, randomized controlled trials with adequate sample sizes—conducted across diverse healthcare systems and sociocultural settings—that utilize standardized outcome definitions, validated measurement tools, and longer follow-up periods will facilitate more reliable effect estimates and enable meta-analyses where appropriate. The limited, low-certainty evidence in areas beyond medication safety—particularly other pediatric clinical domains—highlights the need for further high-quality research.

## 5. Conclusions

This review indicates that interventions incorporating Teach-Back may be beneficial for parents and primary caregivers in pediatric healthcare, particularly for improving medication dosing safety and caregiver medication knowledge. The certainty of evidence was moderate for medication dosing accuracy/safety and low for caregiver medication or safe-dose knowledge. Evidence certainty was low or very low for asthma control, HbA1c levels, oral health/care performance, asthma-related healthcare utilization, diabetes self-care, and caregiver self-efficacy.

Current evidence suggests that interventions incorporating Teach-Back are promising for educating pediatric caregivers; however, because Teach-Back was frequently embedded within multicomponent interventions, its independent contribution remains uncertain, and clinical and methodological heterogeneity limits generalizability across pediatric contexts.

## Figures and Tables

**Table 3:** Characteristics of the included randomized controlled trials.

Study	Country/Setting	Design/N	Population	Teach-Back Intervention	Comparator	Follow-Up	Outcome Domains
Yin et al., 2008 [19]	USA; urban public hospital, pediatric emergency department	Parallel RCT; *N* = 245	Parents/caregivers of children aged 30 days–8 years; liquid medication prescription	HELPix: plain language + patient-specific pictogram-based medication instructions + dose demonstration + Teach-Back + standardized dosing tool	Standard medication counseling	As needed: 3–5 days/end of treatment; mean 12.1 days for observed dosing	Medication knowledge, dosing accuracy, medication administration behavior, and adherence
Chen et al., 2018 [20]	USA; pediatric dentistry	Randomized comparison; *N* = 85	Caregivers of children aged 1–6 years	Teach-Back added to visual-aid counseling to verify toothpaste amount (TB *n* = 43)	Visual-aid counseling	Immediately after education	Appropriate amount of fluoride toothpaste applied/deviation from ideal amount
Ghoneim and Fathalla, 2018 [21]	Egypt; Menoufia University Hospital, pediatrics	RCT; *N* = 60 mother–child dyads (30/30)	Mothers of children with congenital heart disease	Discharge education using Teach-Back; re-teaching of incorrect or incomplete understanding	Routine discharge education	Post-intervention and first follow-up within approximately 1 week	Maternal self-efficacy, understanding/recall of discharge instructions, satisfaction
Li et al., 2020 [11]	USA; large urban emergency department	Parallel RCT; *N* = 149	Parents/guardians of children aged 90 days–11.9 years; acetaminophen and/or ibuprofen	Plain language + simplified handout + unmarked dosing syringe + Teach-Back verifying the correct dose	Standard care	48–72 h and 5–7 days	Knowledge of safe medication dose/correct dosing knowledge
Eakin et al., 2020 [22] *	USA; Baltimore Head Start programs and home setting	Parallel RCT; *N* = 398 (199/199)	Preschool children aged 2–6 years with asthma and their primary caregivers	Asthma Basic Care (ABC) family education + Head Start education; home sessions and supportive calls; closed-loop tell-me-back/show-me-back component	Head Start asthma education alone	3, 6, 9, and 12 months	Asthma control (TRACK), oral corticosteroid use, emergency visits, and hospitalization
Carroll et al., 2024 [5]	USA; tertiary children’s hospital	Parallel RCT; *N* = 198 (99/99)	Caregivers of children aged <=6 years discharged with a new liquid medication prescription	Pictogram-based written medication instructions + structured Teach-Back + dose demonstration + Show-Back	Standard counseling	Approximately 48 h after discharge (2–3 days)	Observed home dosing error and caregiver medication knowledge
Frey et al., 2025 [23]	USA; children’s hospital + home/telehealth	Pilot RCT; *N* = 26	Children aged 5–13 years hospitalized for asthma and their caregivers	TEACHH: pictograms, color/shape-coded medication labels, demonstration, Teach-Back/Show-Back + two Zoom follow-up education sessions	Standard care/standard inpatient asthma education	2–4 days and approximately 3 weeks of education; outcomes at 2, 4, and 6 months	Feasibility, acute healthcare utilization, symptom-free days, caregiver quality of life
Schade et al., 2026 [24]	Brazil; rural southern region	Controlled RCT; *N* = 78	Preschool children and caregivers	Oral hygiene education delivered using the Teach-Back technique	Standard guidance	7 days	Visible Plaque Index (VPI) and Simplified Oral Hygiene Index (OHI-S)
Zelhagh et al., 2026 [25]	Iran; pediatric diabetes care	Three-arm clinical RCT; *N* = 60 (20/20/20)	Children aged 6–12 years with type 1 diabetes and their mothers	Four 30–45 min Teach-Back sessions for mother–child dyads; diet, activity, glucose monitoring, and insulin administration	Multimedia education and routine hospital education	Self-care: 1 month; HbA1c: 3 months	Diabetes self-care behaviors (SDSCA) and HbA1c

* In Eakin et al. [22], the closed-loop “tell-me-back/show-me” strategy was not described in the title or abstract of the main outcomes report; eligibility was confirmed from the trial protocol provided as Supplement 1 to the article. Abbreviations: ABC, Asthma Basic Care; HbA1c, glycated hemoglobin; OHI-S, Simplified Oral Hygiene Index; RCT, randomized controlled trial; SDSCA, Summary of Diabetes Self-Care Activities; TB, Teach-Back; TRACK, Test for Respiratory and Asthma Control in Kids; VPI, Visible Plaque Index. Further details regarding intervention providers, staff training, session duration, the number of Teach-Back cycles, and intervention fidelity or delivery monitoring are provided in Supplementary Table S4. Information on accompanying educational components, comparator conditions, and equivalence of educational attention is presented in Supplementary Table S3. Details not reported in the available study documentation are identified as such rather than assumed.

## Data Availability

No new data were created or analyzed in this study. Data sharing is not applicable to this article.

## Supplementary Materials

The following supporting information can be downloaded at https://www.mdpi.com/article/10.3390/healthcare14213615/s1, Table S1: Database- and Registry-Specific Search Strategies and Search Yields; Table S2: Detailed Risk-of-Bias 2 Assessments and Domain-Level Justifications for the Included Randomized Controlled Trials; Table S3: Components of Teach-Back-Based Interventions and Comparator Attention; Table S4: Intervention Delivery, Staff Training, Teach-Back Cycles, and Implementation Fidelity; Table S5: Outcome-Specific RoB 2–GRADE Linkage and Downgrading Rationale; PRISMA 2020 Checklist.

## Abbreviations

The following abbreviations are used in this manuscript:

ABC	Asthma Basic Care
CENTRAL	Cochrane Central Register of Controlled Trials
CI	Confidence interval
GRADE	Grading of Recommendations Assessment, Development and Evaluation
HbA1c	Glycated hemoglobin
ICTRP	International Clinical Trials Registry Platform
MeSH	Medical Subject Headings
OHI-S	Simplified Oral Hygiene Index
OR	Odds ratio
PRISMA	Preferred Reporting Items for Systematic Reviews and Meta-Analyses
PRISMA-S	Preferred Reporting Items for Systematic Reviews and Meta-Analyses Literature Search Extension
PROSPERO	International Prospective Register of Systematic Reviews
RCT	Randomized controlled trial
RoB 2	Cochrane Risk of Bias 2
RR	Risk ratio
SDSCA	Summary of Diabetes Self-Care Activities
SWiM	Synthesis Without Meta-analysis
TB	Teach-Back
TRACK	Test for Respiratory and Asthma Control in Kids
VPI	Visible Plaque Index
WHO	World Health Organization
