# Supplementary material for: Teach-Back-Based Interventions for Parents and Caregivers in Pediatric Healthcare: A Systematic Review of Randomized Controlled Trials
Source: Healthcare (Basel). 2026 Oct 26;14(21):3615. doi: 10.3390/healthcare14213615 (PMC13763171; doi:10.3390/healthcare14213615)
Supplement: Supplementary file 1 [file healthcare-14-03615-s001.zip › Table S1.pdf]

Supplementary Table S1. Database- and Registry-Specific Search Strategies and Search Yields

Database / platform	Search date (main search)	Search strategy	Records retrieved (n)
**PubMed/MEDLINE**	15 Sep 2026	("Teach-Back Communication"[Mesh]<br>OR "teach-back"[Title/Abstract]<br>OR "teach back"[Title/Abstract]<br>OR teachback[Title/Abstract]<br>OR "show-back"[Title/Abstract]<br>OR "show back"[Title/Abstract]<br>OR (tell[Title/Abstract] AND back[Title/Abstract])<br>OR "teach-to-goal"[Title/Abstract]<br>OR "teach to goal"[Title/Abstract]<br>OR "repeat-back"[Title/Abstract]<br>OR "repeat back"[Title/Abstract]<br>OR "closed-loop communication"[Title/Abstract]<br>OR "closed loop communication"[Title/Abstract]<br>OR "return demonstration"[Title/Abstract]<br>OR (demonstration[Title/Abstract] AND back[Title/Abstract]))<br>AND ("Parents"[Mesh] OR "Caregivers"[Mesh]<br>OR parent*[Title/Abstract] OR caregiver*[Title/Abstract] OR carer*[Title/Abstract]<br>OR mother*[Title/Abstract] OR father*[Title/Abstract] OR guardian*[Title/Abstract] OR famil*[Title/Abstract])<br>AND ("Infant"[Mesh] OR "Child"[Mesh] OR "Adolescent"[Mesh]<br>OR child*[Title/Abstract] OR pediatric*[Title/Abstract] OR paediatric*[Title/Abstract]<br>OR infant*[Title/Abstract] OR newborn*[Title/Abstract] OR neonat*[Title/Abstract]<br>OR toddler*[Title/Abstract] OR preschool*[Title/Abstract] OR adolescen*[Title/Abstract] OR teen*[Title/Abstract])	117
**Cochrane CENTRAL**	15 Sep 2026	#1 [mh "Teach-Back Communication"]<br>#2 ("teach-back" OR "teach back" OR teachback OR "show-back" OR "show back" OR "tell-me-back" OR "tell me back" OR "teach-to-goal" OR "teach to goal" OR "repeat-back" OR "repeat back" OR "closed-loop communication" OR "closed loop communication" OR "return demonstration" OR "demonstration-back" OR "demonstration back"):ti,ab,kw<br>#3 #1 OR #2<br>#4 [mh Parents] OR [mh Caregivers]<br>#5 (parent* OR caregiver* OR carer* OR mother* OR father* OR guardian* OR famil*):ti,ab,kw<br>#6 #4 OR #5<br>#7 [mh Infant] OR [mh Child] OR [mh Adolescent]<br>#8 (child* OR pediatric* OR paediatric* OR infant* OR newborn* OR neonat* OR toddler* OR preschool* OR adolescen* OR teen*):ti,ab,kw<br>#9 #7 OR #8<br>#10 #3 AND #6 AND #9	45

Database / platform	Search date (main search)	Search strategy	Records retrieved (n)
**CINAHL Ultimate (EBSCOhost)**	16 Sep 2026	S1: (MH "Teach-Back Communication") OR TI ("teach-back" OR "teach back" OR teachback OR "show-back" OR "show back" OR "tell-me-back" OR "tell me back" OR "teach-to-goal" OR "teach to goal" OR "repeat-back" OR "repeat back" OR "closed-loop communication" OR "closed loop communication" OR "return demonstration" OR "demonstration-back" OR "demonstration back") OR AB ("teach-back" OR "teach back" OR teachback OR "show-back" OR "show back" OR "tell-me-back" OR "tell me back" OR "teach-to-goal" OR "teach to goal" OR "repeat-back" OR "repeat back" OR "closed-loop communication" OR "closed loop communication" OR "return demonstration" OR "demonstration-back" OR "demonstration back")<br><br>S2: (MH "Parents+") OR (MH "Caregivers+") OR TI (parent* OR caregiver* OR carer* OR mother* OR father* OR guardian* OR famil*) OR AB (parent* OR caregiver* OR carer* OR mother* OR father* OR guardian* OR famil*)<br><br>S3: (MH "Infant+") OR (MH "Child+") OR (MH "Adolescent+") OR TI (child* OR pediatric* OR paediatric* OR infant* OR newborn* OR neonat* OR toddler* OR preschool* OR adolescen* OR teen*) OR AB (child* OR pediatric* OR paediatric* OR infant* OR newborn* OR neonat* OR toddler* OR preschool* OR adolescen* OR teen*)<br><br>S4: S1 AND S2 AND S3	55
**Web of Science Core Collection**	15 Sep 2026	Field: Topic (Web of Science Core Collection; Editions: All)<br><br>("teach-back" OR "teach back" OR teachback OR "show-back" OR "show back" OR "tell-me-back" OR "tell me back" OR "teach-to-goal" OR "teach to goal" OR "repeat-back" OR "repeat back" OR "closed-loop communication" OR "closed loop communication" OR "return demonstration" OR "demonstration-back" OR "demonstration back")<br>AND<br>(parent* OR caregiver* OR carer* OR mother* OR father* OR guardian* OR famil*)<br>AND<br>(child* OR pediatric* OR paediatric* OR infant* OR newborn* OR neonat* OR toddler* OR preschool* OR adolescen* OR teen*)	103
**Scopus**	15 Sep 2026	TITLE-ABS-KEY("teach-back" OR "teach back" OR teachback OR "show-back" OR "show back" OR "tell-me-back" OR "tell me back" OR "teach-to-goal" OR "teach to goal" OR "repeat-back" OR "repeat back" OR "closed-loop communication" OR "closed loop communication" OR "return demonstration" OR "demonstration-back" OR "demonstration back")<br>AND TITLE-ABS-KEY(parent* OR caregiver* OR carer* OR mother* OR father* OR guardian* OR famil*)<br>AND TITLE-ABS-KEY(child* OR pediatric* OR paediatric* OR infant* OR newborn* OR neonat* OR toddler* OR preschool* OR adolescen* OR teen*)	109
**Total records identified from the five bibliographic databases before deduplication**			**429**
**ClinicalTrials.gov**	15 Sep 2026	Teach-Back/closed-loop communication<br>AND<br>parent/caregiver AND pediatric population concepts; no publication-year or language restrictions	21

Database / platform	Search date (main search)	Search strategy	Records retrieved (n)
**WHO ICTRP**	16 Sep 2026	Teach-Back/closed-loop communication<br>AND<br>parent/caregiver<br>AND<br>pediatric population concepts; no publication-year or language restrictions	13
**Total records identified from trial registries before deduplication**			34
**Total records identified from databases and trial registries before deduplication**			463

Additional trial registry searches conducted during revision (14 October 2026)

ClinicalTrials.gov

Search date: 14 October 2026
Search field: Other terms
Exact search string:
("teach-back" OR "teach back" OR teachback OR "show-back" OR "show back" OR "teach-to-goal" OR "closed-loop communication")
Filters: None applied.
Records retrieved: 158.

WHO International Clinical Trials Registry Platform (ICTRP)

Search date: 14 October 2026
Search field: Standard search
Exact search string:
("teach-back" OR "teach back" OR teachback OR "show-back" OR "show back" OR "teach-to-goal" OR "closed-loop communication")
Filters: None applied.
Records retrieved: 173.

Documentation note: These additional searches used a broader Teach-Back terminology strategy without mandatory caregiver or pediatric search blocks to avoid missing relevant studies indexed under adult caregivers or alternative population descriptions. The searches were conducted during manuscript revision and are reported separately from the original September 2026 registry searches. Across the two October exports, 45 records shared identical NCT identifiers. Following eligibility screening of the additional registry records against the review's predefined eligibility criteria, no additional eligible randomized controlled trials were identified. These supplementary searches did not change the nine included studies or the original PRISMA flow diagram.

Additional identification through citation searching: Backward citation searching was conducted by examining the reference lists of included trials and relevant systematic reviews, and forward citation searching was performed for the included trials. Three additional eligible randomized controlled trials were identified and included in the review. The total number of citation records screened was not retained in a separate verifiable screening log; therefore, this number is not reported as a record-identification yield.

Note: No publication-year, language, document-type, or randomized controlled trial filter was applied to the bibliographic database searches. Record counts are raw search yields before deduplication. The five bibliographic databases yielded 429 records, and the two trial registries yielded 34 records, resulting in 463 records in total before deduplication.

Search documentation notes: The PubMed/MEDLINE, CENTRAL, Web of Science, and Scopus yields were verified against the retained final exports/search records. The CINAHL Ultimate search was rerun on 16 September 2026 and reproduced the same 55 records. ClinicalTrials.gov and WHO ICTRP were searched separately and yielded 21 and 13 records, respectively.

Abbreviations: AB, abstract; CENTRAL, Cochrane Central Register of Controlled Trials; MH/MeSH, controlled subject heading; TI, title; kw, keyword; TITLE-ABS-KEY, title/abstract/keywords; WHO ICTRP, World Health Organization International Clinical Trials Registry Platform.
