# Supplementary material for: Teach-Back-Based Interventions for Parents and Caregivers in Pediatric Healthcare: A Systematic Review of Randomized Controlled Trials
Source: Healthcare (Basel). 2026 Oct 26;14(21):3615. doi: 10.3390/healthcare14213615 (PMC13763171; doi:10.3390/healthcare14213615)
Supplement: Supplementary file 1 [file healthcare-14-03615-s001.zip › Table S2.pdf]

Table S2. Detailed Risk-of-Bias 2 Assessments and Domain-Level Justifications for the Included Randomized Controlled Trials

Study / outcome	Domain	Judgment	Brief justification
**Yin et al., 2008 [19] – observed dosing error**	D1	Some concerns	The study was described as randomized, but the available report did not provide sufficient detail on sequence generation and allocation concealment to fully rule out bias from the randomization process.
	D2	Low	The intervention and control conditions were delivered as assigned, with no important deviations from the intended interventions identified that were likely to affect the observed dosing outcome.
	D3	Some concerns	Follow-up was incomplete (227 of 245 randomized caregivers completed follow-up), and observed dosing data were available only for participants contributing the relevant follow-up assessment.
	D4	Low	Dosing accuracy was assessed by direct observation using a predefined error threshold (>20% deviation from the prescribed dose), limiting susceptibility to participant expectations.
	D5	Some concerns	No sufficiently detailed prospective protocol or statistical analysis plan was identified in the available report to confirm that the reported outcome and analysis were selected exactly as prespecified.
	**Overall**	**Some concerns**	**Some concerns were retained because uncertainty remained in the randomization process, missing outcome data, and selection of the reported result, with no domain judged high risk.**
Yin et al., 2008 [19] – incorrect knowledge of daily-dose medication frequency	D1	Some concerns	Random allocation was reported, using sealed envelopes in blocks; however, the methods used to generate the sequence and ensure adequate concealment were incompletely described.
	D2	Low	No important deviations from the assigned counseling strategies were reported that were likely to bias the medication-frequency knowledge comparison (effect of assignment).
	D3	High	The daily-dose medication subgroup initially included 52 intervention and 55 control caregivers, whereas medication-frequency knowledge data were available for only 46 and 53 caregivers, respectively. Missing outcome data were more frequent in the intervention group (6/52 versus 2/55), and missingness could plausibly be related to caregivers' true medication knowledge. The available evidence did not establish that the reported result was robust to the missing data. Based on the outcome-specific RoB 2 signalling-question assessment, this domain was judged to be at high risk of bias.
	D4	Some concerns	Dose-frequency knowledge was elicited in an unblinded follow-up interview; although it was scored against the prescribed dose frequency, awareness of assignment could influence answers or ascertainment.
	D5	Some concerns	Medication knowledge was a designated secondary domain and multiple knowledge endpoints were reported, but an adequately detailed dated statistical analysis plan for this specific contrast was not verified.

Study / outcome	Domain	Judgment	Brief justification
	Overall	High	The overall high-risk-of-bias judgment was driven by the high risk arising from missing outcome data (D3). Additional concerns were identified regarding the randomization process (D1), measurement of caregiver-reported medication knowledge (D4), and selection of the reported result (D5), while deviations from intended interventions (D2) were judged to be at low risk. This assessment applies specifically to medication-frequency knowledge and does not change the separate RoB 2 assessment for observed medication dosing errors.
**Chen et al., 2018 [20] – deviation from ideal toothpaste amount**	D1	Some concerns	Caregivers were reported to be randomly assigned to Teach-Back or visual-aid counseling, but the method of sequence generation and allocation concealment was not described in sufficient detail.
	D2	Low	The randomized educational conditions were implemented as intended, and no important intervention-related deviations were identified.
	D3	Low	The outcome was assessed immediately after education and the reported analysis did not indicate a material amount of missing outcome data.
	D4	Low	Toothpaste quantity was measured by weighing each application, providing an objective assessment of deviation from the ideal amount.
	D5	Some concerns	A prospective protocol or analysis plan was not available in the report to verify that the reported outcome definition and analysis were fully prespecified.
	**Overall**	**Some concerns**	**The objective outcome measurement and minimal missing-data concern supported low risk in D2–D4, while incomplete reporting of randomization and prespecification resulted in some concerns overall.**
**Ghoneim & Fathalla, 2018 [21] – maternal self-efficacy**	D1	Some concerns	The report states that a simple random sample was divided into two equal groups, but the random sequence generation and allocation-concealment procedures were not clearly described.
	D2	Low	No important deviations from the assigned discharge-education conditions were reported that would be expected to materially bias the self-efficacy comparison.
	D3	Some concerns	The report estimated a sample of 74 but analyzed 60 mother–child dyads after 14 children were excluded because of referral to surgery or death; the timing of these exclusions relative to randomization and their handling were not fully clear.
	D4	High	Maternal self-efficacy was self-reported, and participants were aware of the educational intervention received; the outcome was therefore susceptible to knowledge of intervention assignment.
	D5	Some concerns	No prospective trial registration, protocol, or statistical analysis plan was identified to verify prespecified outcome selection and analysis across the reported time points.
	**Overall**	**High**	**The high risk of bias in measurement of the self-reported self-efficacy outcome determined the overall high-risk judgment.**

Study / outcome	Domain	Judgment	Brief justification
**Li et al., 2020 [11] – safe dose knowledge at 48–72 h**	D1	Some concerns	The trial was randomized, but the main report provided limited detail on sequence generation and allocation concealment, and the randomized groups were unequal in size (66 intervention, 83 control).
	D2	Low	Treating clinicians were not informed of intervention components or assignment, and no important deviations from the intended educational conditions were identified.
	D3	High	Only 97 of 149 randomized participants (65%) were reached for the primary 48–72 h follow-up; substantial loss to follow-up and the unequal numbers contributing outcome data created a high risk of bias from missing outcome data.
	D4	Some concerns	The primary outcome was caregiver-reported safe-dose knowledge during follow-up calls. Participants and the research assistant could not be blinded, so reporting or assessment could have been influenced by intervention awareness.
	D5	Low	The trial was prospectively registered (NCT03223246), and the primary 48–72 h safe-dosing outcome and primary hypothesis were clearly specified.
	**Overall**	**High**	**The substantial amount of missing primary-outcome data resulted in an overall high-risk judgment despite low risk for deviations and selective reporting.**
**Eakin et al., 2020 [22] – TRACK asthma control**	D1	Low	A statistician generated the 1:1 block-randomization sequence using a random-number generator, with assignments placed in sealed envelopes opened after completion of baseline assessments.
	D2	Low	Analyses followed the intention-to-treat principle and sensitivity analyses were reported; no important deviations from randomized assignment were identified as likely to bias the effect estimate.
	D3	Some concerns	Longitudinal follow-up was incomplete (e.g., TRACK data were available for 310 of 398 randomized participants at 12 months), creating some concern about missing outcome data.
	D4	Some concerns	TRACK is a caregiver-reported asthma-control measure. Outcome assessors were blinded, but caregivers were aware of the intervention received, so responses could potentially have been influenced by assignment.
	D5	Some concerns	The report states that the original primary outcome (symptom-free days) was changed to TRACK after a data safety and monitoring board recommendation during the study, creating some concern about selection of the reported result despite transparent reporting of the change.
	**Overall**	**Some concerns**	**The trial had strong randomization procedures, but missing follow-up data, participant-reported measurement, and the documented primary-outcome change supported an overall judgment of some concerns.**
**Eakin et al., 2020 [22] – Asthma-related hospitalization**	D1	Low	A statistician generated the block-randomization sequence, and assignments were concealed in sealed envelopes until baseline assessments were completed.
	D2	Low	No important deviations from the assigned educational interventions were identified as likely to bias the effect of assignment on hospitalization.

Study / outcome	Domain	Judgment	Brief justification
	D3	Some concerns	Hospitalizations were ascertained through caregiver reports collected during follow-up. Follow-up was incomplete, and the number of participants contributing specifically to the hospitalization analysis could not be established from the reported information.
	D4	Some concerns	Hospitalizations were caregiver-reported rather than verified against medical records. Although research assistants were blinded, caregivers were aware of their group assignment, leaving some potential for reporting bias.
	D5	Some concerns	Healthcare utilization was a planned secondary outcome, but the prespecification of the specific hospitalization analysis could not be fully verified against a detailed, dated analysis plan.
	**Overall**	**Some concerns**	**Incomplete outcome ascertainment, caregiver-reported measurement, and uncertainty regarding the prespecification of the analysis supported the overall judgment of some concerns.**
**Carroll et al., 2024 [5] – observed home dosing error**	D1	Low	Caregiver–child dyads were assigned 1:1 using permuted-block randomization, and the study protocol was available.
	D2	Low	No important deviations from the intended communication bundle or standard counseling were identified that were likely to bias the primary outcome.
	D3	Some concerns	The primary observed-dosing outcome was available for 151 of 198 randomized caregivers (76.3%). Although completers and non-completers did not differ on reported baseline characteristics, the amount of missing outcome data warranted some concern.
	D4	Low	The primary outcome was based on caregiver-submitted photographs of medication-filled syringes and was independently measured by two trained reviewers who were blinded to group assignment.
	D5	Low	The trial was registered (NCT05143047), a protocol was available, and the primary outcome and main analysis were prespecified.
	**Overall**	**Some concerns**	**The main concern was incomplete primary-outcome ascertainment; the other domains were judged low risk.**
Carroll et al., 2024 [5] – correct caregiver-reported medication dose	D1	Low	Permuted-block random allocation was described and a trial protocol is available; no clear evidence of failure of randomization was identified.
	D2	Low	The communication intervention and standard counseling were delivered according to allocation. Encouraged use of the discharge instruction sheet was part of the assigned intervention, not a protocol deviation.
	D3	Some concerns	The medication-knowledge survey was completed by 76/99 intervention and 69/99 control caregivers; missing responses could depend on unobserved medication knowledge.
	D4	Some concerns	Caregiver-reported correct dose was scored against the prescription, but participants were aware of allocation and intervention caregivers were encouraged to consult an instruction sheet during the survey.

Study / outcome	Domain	Judgment	Brief justification
	D5	Some concerns	Medication-dose knowledge was a prespecified secondary outcome, but the precise prespecified analysis of this dichotomized contrast could not be fully confirmed against a dated plan.
	Overall	Some concerns	The three authors independently agreed on some concerns overall. D3, D4 and D5 create uncertainties, but no individual domain was rated high; the authors judged that their combined effect did not reach high risk overall.
**Frey et al., 2025 [23] – symptom-free days at 6 months**	D1	Low	The study was a registered randomized pilot trial (NCT04995692), with random allocation and concealed allocation reported in available trial-method assessments.
	D2	Low	The inpatient intervention was completed for all intervention participants and the trial did not identify important deviations from assigned care likely to bias the symptom outcome.
	D3	High	Follow-up for caregiver-reported symptom-free days was substantially incomplete; at 6 months, outcome data were available for 7 intervention and 9 control participants out of 26 randomized participants.
	D4	Some concerns	Symptom-free days were reported by caregivers. Follow-up surveys were collected with blinded assessment procedures, but caregivers knew the intervention received, leaving some potential for reporting bias.
	D5	Low	The trial was prospectively registered, and symptom-free days at 2, 4, and 6 months were prespecified outcomes.
	**Overall**	**High**	**Substantial missing outcome data at 6 months determined the overall high-risk judgment.**
**Frey et al., 2025 [23] – Asthma-related acute healthcare use**	D1	Low	Randomization used permuted blocks, with the allocation sequence developed independently by a biostatistician and implemented electronically through REDCap.
	D2	Low	No important deviations from the assigned interventions were identified as likely to bias the effect of assignment on acute healthcare use.
	D3	Low	Acute healthcare utilization was assessed using electronic health records, allowing events to be identified independently of caregiver survey completion and reducing concerns about missing outcome data.
	D4	Low	Asthma-related emergency department visits and hospitalizations were identified from electronic health records using the same approach for both groups, limiting the potential for differential outcome measurement.
	D5	Some concerns	Acute healthcare utilization was a planned outcome. However, the detailed prespecification of the final analysis and reporting approach could not be fully verified.
	**Overall**	**Some concerns**	**The remaining uncertainty concerned the prespecification of the reported analysis; the other domains were judged to be at low risk of bias.**
**Schade et al., 2026 [24] – 7-day visible plaque index**	D1	Low	Participants were assigned in blocks of four using a random-number sequence generated in Microsoft Excel; allocation was performed by a researcher who remained blinded to the intervention type.

Study / outcome	Domain	Judgment	Brief justification
	D2	Some concerns	Blinding of caregivers and intervention delivery personnel was not feasible for the educational comparison, and the available report did not fully exclude deviations related to awareness of assigned intervention.
	D3	Some concerns	The final analyzed sample was smaller than the planned target, and the available reporting did not fully resolve the potential influence of missing or unavailable outcome data on the 7-day analysis.
	D4	Low	Visible plaque was assessed using a clinical plaque index rather than a participant-reported outcome, reducing susceptibility to participant expectations.
	D5	Some concerns	Although the trial was prospectively registered and the visible plaque outcome was prespecified, a sufficiently detailed prospective analysis plan was not available to confirm that the specific psychosocial subgroup/effect-modification analyses were fully prespecified.
	**Overall**	**Some concerns**	**Some concerns remained regarding deviations from intended interventions, missing outcome data, and prespecification of the subgroup analyses; however, no domain was judged to be at high risk of bias.**
**Zelhagh et al., 2026 [25] – SDSCA self-care**	D1	Low	The three-arm clinical trial reported randomized allocation, with no material baseline imbalance identified that suggested a failure of randomization.
	D2	Low	No important deviations from the assigned Teach-Back, multimedia, or routine-education conditions were identified as likely to bias the self-care comparison.
	D3	Low	The reported self-care analysis included the randomized groups without a material amount of missing outcome data being evident in the study report.
	D4	High	SDSCA self-care was a participant-reported behavioral outcome, and participants were aware of the educational approach received; responses could therefore have been influenced by knowledge of intervention assignment.
	D5	Some concerns	No sufficiently detailed prospective protocol or statistical analysis plan was available to fully exclude selective choice among possible self-care analyses or time points.
	**Overall**	**High**	**High risk in measurement of the self-reported SDSCA outcome determined the overall high-risk judgment.**
**Zelhagh et al., 2026 [25] – HbA1c**	D1	Low	The three-arm clinical trial reported randomized allocation, with no material baseline imbalance identified that suggested a failure of randomization.
	D2	Low	No important deviations from the assigned educational conditions were identified as likely to bias the HbA1c comparison.
	D3	Low	The reported HbA1c analysis did not indicate a material amount of missing outcome data across randomized groups.
	D4	Low	HbA1c is an objective laboratory measure and is unlikely to be influenced by participant or assessor awareness of intervention assignment.

Study / outcome	Domain	Judgment	Brief justification
	D5	Some concerns	The trial was retrospectively registered (IRCT20231128060208N1), with self-care and HbA1c outcomes listed in the registry. The registered sample size was 45 participants, whereas 60 participants were reported in the publication. A sufficiently detailed, dated statistical analysis plan specifying the reported comparisons was not identified. These limitations support some concerns regarding selection of the reported result.
	**Overall**	**Some concerns**	**The objective HbA1c measurement supported low risk in D4; the remaining concern related to prespecification and selection of the reported result.**

Note. RoB 2 judgments are outcome-specific. D1, bias arising from the randomization process; D2, bias due to deviations from intended interventions; D3, bias due to missing outcome data; D4, bias in measurement of the outcome; D5, bias in selection of the reported result.
