# Supplementary material for: Teach-Back-Based Interventions for Parents and Caregivers in Pediatric Healthcare: A Systematic Review of Randomized Controlled Trials
Source: Healthcare (Basel). 2026 Oct 26;14(21):3615. doi: 10.3390/healthcare14213615 (PMC13763171; doi:10.3390/healthcare14213615)
Supplement: Supplementary file 1 [file healthcare-14-03615-s001.zip › Table S3.pdf]

Supplementary Table S3. Teach-Back intervention components, comparator conditions, and educational attention in included randomized trials

Study	Teach-Back / closed-loop feature	Other components and reported dose	Comparator	Educational attention and interpretation
Yin et al., 2008 [19]	Caregiver demonstrated intended liquid-medication dose back to study staff after dosing demonstration.	HELPix plain-language, 2-page pictogram medication sheets and log; dosing demonstration and standardized oral syringe. Entire counseling/Teach-Back contact: 1.5–3 min.	Routine medication counseling by clinical staff; standardized dosing tool not routinely supplied.	Control contact duration not reported; no time-matched comparator documented. Multiple educational elements differed.
Chen et al., 2018 [20]	After shared visual-aid counseling, the TB arm received feedback on the weighed toothpaste dose and reapplied a corrected amount (43 assigned).	Both arms: ADA smear/pea visual aids, brief fluorosis-risk explanation, age-based dosing guidance, and weighed application. An interview script standardized delivery; formal fidelity outcomes not reported.	Visual-aid counseling alone, with an additional weighed application but without the subsequent TB feedback/reapplication.	Instruction time per arm not reported. The TB arm received an additional feedback-guided application; equal educational attention was not established.
Ghoneim & Fathalla, 2018 [21]	Closed-loop verification of mothers' comprehension during discharge education.	Nurses received Teach-Back implementation training: four 30–45-min training sessions (for NURSES, not caregiver contact). Mother-facing intervention time not reported.	Routine discharge instructions to mothers.	Mother-facing contact duration not reported in full article; not an established attention-matched comparison.
Li et al., 2020 [11]	Safe-dose understanding checked through caregiver syringe demonstration/Teach-Back (1–3 cycles reported).	Additional brief researcher-led education after usual discharge: simplified weight-specific handout; syringe and dosing demonstration.	Usual ED discharge teaching alone; study follow-up calls applied to both arms.	Added teaching occurred only in intervention arm; exact comparative duration not reported, so attention not matched.
Eakin et al., 2020 [22]	Tell-me-back/show-me strategy as part of tailored Asthma Basic Care (ABC) family education.	In addition to Head Start activities: four 30–45-min home education sessions with iPad materials, problem solving and goal setting; three booster telephone calls.	Head Start asthma education and community program activities alone.	Clearly additional intervention-specific contacts (four planned home sessions and three calls); not an attention-matched comparison.
Carroll et al., 2024 [5]	Structured Teach-Back and syringe-dose Show-Back at hospital discharge.	Patient-specific plain-language pictogram sheet; staff dosing demonstration and graduated syringe; mean bundled-intervention time 5 min 9 sec (SD 4 min 39 sec).	Routine discharge counseling and EHR-generated instructions; graduated dosing syringe also supplied to intervention arm.	Routine counseling occurred in both arms, but extra package was intervention-specific; standard-counseling time not reported.
Frey et al., 2025 [23]	Teach-Back/Show-Back during hospital education and remote reinforcement.	Pictorial action plan, coded labels and demonstrations; two Zoom visits (days 2–4 and ~3 weeks later).	Standard inpatient asthma education and usual post-discharge care.	Intervention added inpatient teaching and two planned telehealth contacts; no matched educational attention control.
Schade et al., 2026 [24]	Teach-Back after same verbal oral-hygiene teaching; re-explanation up to 3 loops plus caregiver return demonstration of toothbrushing.	Both arms received same teaching content, 14 laminated graphic sheets and toothbrushing model demonstration during home visit; oral-hygiene kit for all.	Same oral-health education/materials and toothbrushing demonstration but without Teach-Back or return demonstration.	Content/materials shared across arms; a relatively focused Teach-Back contrast, but actual educational contact time by group not reported.
Zelhagh et al., 2026 [25]	Interactive teach–check–reteach, with previous content reviewed at subsequent sessions.	Individual diabetes self-care teaching; mean four 30–45-min sessions (daily); education booklet provided after first session.	Multimedia group: five mobile videos/files covering same topics; routine-care group: usual staff education and posters.	Comparable topics in Teach-Back and multimedia groups, but video viewing time and educator contact not reported; routine care not time matched.

Educational attention refers to intervention-specific instructional contact, not research outcome assessments. "Not reported" denotes that the source available for verification did not provide comparable contact-time information; it does not prove equal or unequal duration. Only contrasts sharing other educational content permit a more focused assessment of the additional Teach-Back component.
