# Supplementary material for: Teach-Back-Based Interventions for Parents and Caregivers in Pediatric Healthcare: A Systematic Review of Randomized Controlled Trials
Source: Healthcare (Basel). 2026 Oct 26;14(21):3615. doi: 10.3390/healthcare14213615 (PMC13763171; doi:10.3390/healthcare14213615)
Supplement: Supplementary file 1 [file healthcare-14-03615-s001.zip › Table S4.pdf]

Supplementary Table S4. Delivery, staff training, Teach-Back cycles, and implementation fidelity

Study	Educator and staff training	Dose, session duration and Teach-Back cycles	Implementation fidelity / delivery monitoring
**Yin et al., 2008 [19]**	Trained and supervised research assistants (supervision by trial investigator).	Single ED-discharge counseling plus demonstration/Teach-Back; 1.5–3 min for entire intervention; number of corrective cycles NR.	Staff training/supervision stated; no quantified observation checklist or intervention-adherence rate reported.
**Chen et al., 2018 [20]**	Person delivering caregiver instruction not separately identified; interviewer used a standardized script.	Single encounter. Shared visual-aid instruction, then one additional feedback-guided toothpaste reapplication in the TB arm; minutes and repeated TB cycles NR.	Interview script intended to standardize delivery; no independent fidelity assessment/results reported.
**Ghoneim & Fathalla, 2018 [21]**	Pediatric nurses; 40 nurses divided into four groups for research-led in-service training (four 30–45-min teaching sessions reported).	Mother-facing discharge Teach-Back; duration and number of correction cycles NR. Nurse training time MUST NOT be described as caregiver exposure.	Six-item observational Teach-Back Discharge Education Audit evaluated nurse skills; no per-session protocol-adherence rate provided.
**Li et al., 2020 [11]**	Bilingual premedical research assistant, trained in the trial protocol by two investigators.	Additional brief ED-discharge session; repeat dose demonstration until correct (1–3 TB cycles); 48/64 (75%) demonstrated correctly on first attempt; minutes NR.	Standardized script/checklist; RA observed monthly for first 3 months and quarterly thereafter for intervention consistency.
**Eakin et al., 2020 [22]**	Trained Head Start staff or Johns Hopkins asthma educators; yearly Head Start staff asthma training also offered.	Four 30–45-min home education sessions plus three booster telephone calls; number of individual tell-me-back/show-me loops NR.	Interventionist training and exposure-based as-treated analyses documented; no numerical Teach-Back fidelity/adherence assessment reported in main report.
**Carroll et al., 2024 [5]**	Study PI (pediatric hospitalist) or trained research assistant.	Single discharge communication bundle; mean delivery 5 min 9 sec (SD 4 min 39 sec). Further explanation provided if needed; cycles NR.	Scripted/standardized materials and trained personnel; no separate quantified intervention-delivery fidelity audit reported.
**Frey et al., 2025 [23]**	Dedicated asthma educator delivered inpatient and virtual instruction; sessions used scripted educational materials.	One inpatient session and two Zoom follow-ups (2–4 days and ~3 weeks); TB/Show-Back counts and session length NR.	Protocol: session checklists and ≥20% recording/review planned; published report: 100% inpatient teaching and 77% virtual visits completed. Verification of planned recordings NR.
**Schade et al., 2026 [24]**	Two previously trained researchers delivered home visits; one researcher specifically trained in five-step TB.	Single home education visit; identical 14 pictorial sheets in both groups; re-teach/check cycle up to three repetitions, with caregiver return demonstration; session length NR.	Ten-family pilot refined content/language; provider training documented; session-level independently scored fidelity NR.
**Zelhagh et al., 2026 [25]**	Individual child–mother education; professional identity and specific interventionist training not clearly stated.	Mean four individual 30–45-min sessions, approximately daily; open-ended comprehension checks and re-teaching as needed; number of repetitions NR.	Re-explanation/review described in intervention protocol; no separate scored fidelity or adherence rate reported.

Abbreviations: TB, Teach-Back; ED, emergency department; RA, research assistant; PI, principal investigator; NR, not reported or not verifiable in the consulted original materials. Completion of scheduled sessions does not establish fidelity to individual Teach-Back steps.

Source qualification: For Frey [23], the ≥20% recording/review requirement is protocol-specified (ClinicalTrials.gov NCT04995692), not a verified completion percentage in the published report. For Ghoneim & Fathalla [21], 30–45-minute sessions trained nurses, not caregivers. The absence of fidelity data should not be interpreted as evidence that fidelity monitoring did not occur.
