# Supplementary material for: Teach-Back-Based Interventions for Parents and Caregivers in Pediatric Healthcare: A Systematic Review of Randomized Controlled Trials
Source: Healthcare (Basel). 2026 Oct 26;14(21):3615. doi: 10.3390/healthcare14213615 (PMC13763171; doi:10.3390/healthcare14213615)
Supplement: Supplementary file 1 [file healthcare-14-03615-s001.zip › Table S5.pdf]

## Supplementary Table S5. Outcome-Specific RoB 2–GRADE Linkage and Downgrading Rationale

| Critical GRADE domain | Outcome-specific RoB 2 assessments (overall judgment) | GRADE downgrades | Certainty |
|---|---|---|---|
| Medication dosing accuracy/safety [5,19] | Yin—observed dosing error: some concerns; Carroll—observed home dosing error: some concerns | RoB −1; inconsistency 0; indirectness 0; imprecision 0 | MODERATE |
| Caregiver medication/safe-dose knowledge [5,11,19] | Yin—incorrect daily-dose frequency knowledge: high; Li—safe-dose knowledge: high; Carroll—correct reported dose: some concerns | RoB −1; inconsistency 0; indirectness 0; imprecision −1 | LOW |
| Oral health/care performance [20,24] | Chen—toothpaste deviation: some concerns; Schade—visible plaque index: some concerns | RoB −1; inconsistency −1; indirectness 0; imprecision −1 | VERY LOW |
| Asthma control (TRACK) [22] | Eakin—TRACK: some concerns | RoB −1; inconsistency not assessable; indirectness 0; imprecision −1 | LOW |
| Asthma-related healthcare utilization [22,23] | Eakin—hospitalization: some concerns; Frey—acute care use from health records: some concerns | RoB −1; inconsistency −1; indirectness 0; imprecision −1 | VERY LOW |
| Diabetes self-care [25] | Zelhagh—SDSCA: high | RoB −2; inconsistency not assessable; indirectness 0; imprecision −1 | VERY LOW |
| HbA1c [25] | Zelhagh—HbA1c: some concerns | RoB −1; inconsistency not assessable; indirectness 0; imprecision −1 | LOW |
| Caregiver self-efficacy [21] | Ghoneim and Fathalla—maternal self-efficacy: high | RoB −2; inconsistency not assessable; indirectness 0; imprecision −1 | VERY LOW |

**Interpretation of downgrading decisions**

**Medication dosing accuracy/safety [5,19]:** Risk of bias −1: neither direct-observation result was judged at low overall RoB 2 risk (Yin and Carroll, both some concerns). Inconsistency 0: no clearly conflicting direction for the two observed dosing outcomes. Indirectness 0: caregiver dosing tasks directly matched the review question. Imprecision 0: available direct-observation data showed clinically relevant reductions, although the studies were not pooled.

**Caregiver medication/safe-dose knowledge [5,11,19]:** Risk of bias −1: Outcome-specific RoB 2 assessments identified high risk of bias for Li et al. and Yin et al., primarily related to missing outcome data, and some concerns for Carroll et al. related to incomplete follow-up responses, outcome measurement, and reporting. These limitations were considered serious for the body of evidence. Although they could materially influence individual findings, the available studies provided evidence of improvements in selected, but not all, medication-knowledge outcomes. Given the heterogeneous outcome definitions and the absence of a pooled effect estimate, the findings were interpreted cautiously, and the risk-of-bias limitations were judged to warrant a one-level downgrade. Imprecision −1: The available outcome-specific analyses involved limited numbers of participants, with uncertainty regarding the precision and clinical importance of the reported effects. Inconsistency 0; indirectness 0. Overall GRADE certainty: LOW.

**Oral health/care performance [20,24]:** Risk of bias −1: Chen and Schade both had some concerns; the favorable Schade finding was subgroup-dependent. Inconsistency −1: no clear additional mean-deviation effect in Chen versus a conditional plaque-index signal in Schade, with differing measures. Imprecision −1: only 163 participants across small, nonpooled trials and uncertain subgroup effects. Indirectness 0: both measured pediatric oral-care performance.

**Asthma control (TRACK) [22]:** Risk of bias −1: Eakin TRACK had some concerns related to follow-up, caregiver reporting, and analysis selection. Imprecision −1: the three-month effect estimate ($\beta = 6.26$; 95% CI 1.77–10.75) spanned small and potentially important differences. Inconsistency not assessable (one study); indirectness 0 (direct pediatric asthma-control outcome).

**Asthma-related healthcare utilization [22,23]:** Risk of bias −1: Eakin hospitalization and Frey record-based acute healthcare use had some concerns overall, arising in different domains. Inconsistency −1: effect patterns and utilization definitions differed across the large trial and small feasibility RCT. Imprecision −1: Frey included only 26 participants with sparse events, preventing a reliable comparative estimate. Indirectness 0: both assessed acute asthma-related care use.

**Diabetes self-care [25]:** Risk of bias −2: Zelhagh SDSCA self-care was rated high overall because this self-reported outcome was susceptible to measurement bias in an unblinded education trial. Imprecision −1: one small three-arm trial (60 randomized, 20 per arm) and uncertain robustness. Inconsistency not assessable (one study); indirectness 0 (targeted self-care behaviors).

**HbA1c [25]:** Risk of bias −1: the HbA1c assessment had some concerns about selection/prespecification of the reported analysis. Imprecision −1: evidence was based on a single small three-arm randomized trial (20 participants per group), limiting confidence in the precision and robustness of the between-group comparisons. Inconsistency not assessable; indirectness 0 (direct biochemical measure). The unusually large HbA1c reductions reported in both active intervention groups warrant cautious interpretation, particularly because concurrent treatment changes were insufficiently characterized.

**Caregiver self-efficacy [21]:** Risk of bias −2: Ghoneim and Fathalla maternal self-efficacy was rated high overall, especially for subjective measurement without participant blinding and uncertainty about missing outcomes. Imprecision −1: only one small randomized trial (60 dyads), without an adequately precise comparative effect estimate. Inconsistency not assessable; indirectness 0 for caregiver self-efficacy.

Additional outcomes (not independent inputs to Table 3 GRADE ratings): Frey et al. [23] symptom-free days at 6 months was assessed with RoB 2 and judged high overall risk because of substantial survey attrition; Eakin et al. [22] oral corticosteroid courses was described narratively as a supporting result but was not assigned a separate outcome-specific RoB 2 judgment in Table 2. Their results were not used to determine the eight reported GRADE domain ratings.

Abbreviations: RoB, risk of bias; GRADE: Grading of Recommendations Assessment, Development and Evaluation; RCT, randomized controlled trial; TRACK, Test for Respiratory and Asthma Control in Kids; SDSCA, Summary of Diabetes Self-Care Activities. Zero denotes no GRADE downgrade; negative integers indicate the number of certainty levels lowered in that domain. For one-trial domains, inconsistency cannot be evaluated and was not used as a reason for downgrading. Publication bias was not formally assessed and was not automatically downgraded.
